# The *Agrobacterium fabrum* efflux pump PecM serves as a critical metabolic relief valve to maintain cellular homeostasis

**DOI:** 10.1128/msphere.00601-26

**Published:** 2026-10-08

**Authors:** Arpita Ghosh, Ahmed Al-Tohamy, Fabrizio Donnarumma, Anne Grove

**Affiliations:** 1 Department of Biological Sciences, Louisiana State University 124526, Baton Rouge, Louisiana, USA; 2 Department of Chemistry, Louisiana State University 213879, Baton Rouge, Louisiana, USA; Kansas State University, Manhattan, Kansas, USA

**Keywords:** oxidative stress, mass spectrometry, efflux pumps

## Abstract

*Agrobacterium fabrum* infects a wide range of plants, resulting in significant economic losses. In the rhizosphere, the bacterium is exposed to several stressors, including compounds secreted from other bacteria, fungi, or plants. *A. fabrum* encodes the efflux pump PecM for which the substrate is 4-hydroxybenzaldehyde, a compound that is both present in plant exudates and generated by bacteria during degradation of aromatic compounds. As an aromatic aldehyde, 4-hydroxybenzaldehyde is a chemically reactive electrophile that can react with cellular nucleophiles such as thiol groups on proteins and glutathione, leading to disruption of redox homeostasis. To assess the physiological consequences of *pecM* disruption, we compared the transcriptome and proteome profiles of wild-type and *pecM* disruption strains. While 30 genes were seen to be differentially expressed in the *pecM* disruption strain, 118 proteins differentially accumulated. Accumulating proteins included glutathione S-transferase and glutathione-dependent peroxiredoxin, both of which protect from oxidative stress. Consistent with this observation, the *pecM* disrupted strain was more resistant to oxidative stress. Across the genes and proteins detected in both data sets, there was no overall linear relationship, as reflected in a Pearson correlation of ~0. A marked depletion of bS21C, which is a component of the small ribosomal subunit, was observed; as one of three paralogs, its differential expression suggests the possibility that ribosome heterogeneity is part of adaptation to stress. Taken together, our data suggest that absence of PecM imposes a 4-hydroxybenzaldehyde-induced oxidative or electrophile stress and that PecM serves as a critical metabolic relief valve to maintain cellular homeostasis.

**IMPORTANCE:**

Efflux pumps often receive attention in the context of protecting bacteria from environmental toxins such as antibiotics. However, they are also important for exporting metabolic intermediates that may otherwise disrupt cellular processes. Bacterial metabolism of aromatic compounds can generate 4-hydroxybenzaldehyde, which the bacteria may also encounter in the rhizosphere, and its intracellular accumulation would be expected to disrupt redox homeostasis. Here, we show that disruption of the *Agrobacterium fabrum* efflux pump PecM, which exports 4-hydroxybenzaldehyde, results in widespread proteomic remodeling, with marked accumulation of proteins associated with oxidative and electrophile stress. These findings emphasize the importance of efflux systems in preventing the accumulation of reactive intermediates, and they expand our understanding of how such efflux systems contribute to metabolic balance and global cellular adaptation in complex environmental niches such as the rhizosphere.

## INTRODUCTION

Microbes residing in the soil are challenged by several biotic and abiotic factors. The rhizosphere is a particularly competitive environment as plants and other microbes synthesize and secrete a variety of compounds, including antimicrobial peptides and secondary metabolites intended to kill would-be pathogens or competitors (1). Bacteria inhabiting the rhizosphere must, therefore, adapt to the dynamic changes in this ecological niche to survive. In many instances, exposure to toxic compounds triggers the expression of bacterial genes encoding efflux pumps. Efflux pumps are capable of extruding a wide range of structurally different compounds, such as antibiotics, detergents, solvents, and heavy metals, and they may even confer multidrug resistance (2). In other circumstances, efflux pumps may serve to relieve the cells of potentially toxic metabolic intermediates (3, 4).

*Agrobacterium fabrum* is a Gram-negative α-Proteobacterium of the family *Rhizobiaceae* and the causative agent of crown gall disease in hundreds of plant species, primarily dicotyledonous plants (5). To establish infection, the bacteria transfer a specific piece of DNA termed the T-DNA from the tumor-inducing plasmid into the host genome, with the T-DNA-encoded proteins modulating the expression of plant genes, leading to the formation of the characteristic tumor (5, 6). The process of tumorigenesis and the proteins associated with this process have been extensively studied; based on this knowledge, *A. fabrum*, which was previously classified as *Agrobacterium tumefaciens* genomovar G8, has become a critical tool for plant engineering.

In *A. fabrum*, transcription factor PecS has been shown to control phenotypes associated with bacterial fitness and survival in the rhizosphere (7). PecS is a member of the multiple antibiotic resistance regulator (MarR) protein family, which binds a small-molecule ligand or oxidant, as a result of which cognate genes are differentially expressed (8, 9). The *pecS* gene is divergent to *pecM*, which codes for an efflux pump belonging to the drug/metabolite exporter (DME) family, one of the largest families within the drug/metabolite transporter (DMT) superfamily. The expression of both *pecS* and *pecM* is repressed by PecS (7, 10). The *pecS-pecM* gene pair was first characterized in *Dickeya dadantii*, the causative agent of soft rot disease in plants (11). In *D. dadantii*, PecS controls many genes, including the expression of pectinase genes that are critical for infectivity and disease progression, and it also limits the biosynthesis of indigoidine, a blue pigment, which acts as an antioxidant and is exported by *D. dadantii* PecM (12). The *pecS-pecM* gene pair has also been investigated in members of the γ-proteobacterial species *Pectobacterium*, *Klebsiella*, and *Vibrio* and in the actinobacterial genus *Streptomyces* (13–17). Besides *Dickeya* species, other bacteria that harbor the *pecS-pecM* locus do not encode indigoidine biosynthetic genes; hence, the substrate(s) of their PecM proteins must be different. For *A. fabrum* PecM, the substrate was recently shown to be the cellular metabolite and plant exudate 4-hydroxybenzaldehyde (4HBA), as evidenced in part by its accumulation in a *pecM* disruption strain (18). A very marked induction of *pecM* expression occurs in the presence of 4HBA, further supporting a physiological role for PecM in extruding this compound (18).

Generally, aldehydes such as 4HBA are electrophilic, albeit with variable reactivity (19). Aromatic aldehydes can form adducts with proteins and thiols, and their toxicity is typically averted by their conversion to the corresponding acids (20). 4HBA is found in plant exudates, where it may derive from lignin degradation, and it may be produced as an intermediate in microbial degradation of aromatic compounds, such as tyrosine or lignin monomers (21–24). Considering that 4HBA accumulates as a metabolic intermediate in an *A. fabrum pecM* disruption strain (18) and given that the bacteria are exposed to this compound in the rhizosphere, we investigated the role of PecM in *A. fabrum* physiology by comparing wild-type (WT) and *pecM* disruption strains using a combination of transcriptomics and quantitative proteomics. We show here that the phenotypic consequences of *pecM* disruption entail increased resistance to oxidative stress and that maintenance of cellular homeostasis in the absence of PecM requires very extensive cellular and metabolic remodeling.

## RESULTS AND DISCUSSION

### PecM substrates

The distribution of the *pecS-pecM* gene pair was previously reported to be sporadic, with the genes primarily found in Proteobacteria (Pseudomonadota) and in some members of the genus *Streptomyces* (13). While *pecS-pecM* is encoded by several members of the α-Proteobacteria, to which *A. fabrum* belongs, the genomic context appears not to be highly conserved (Fig. S1).

**Fig 1:**
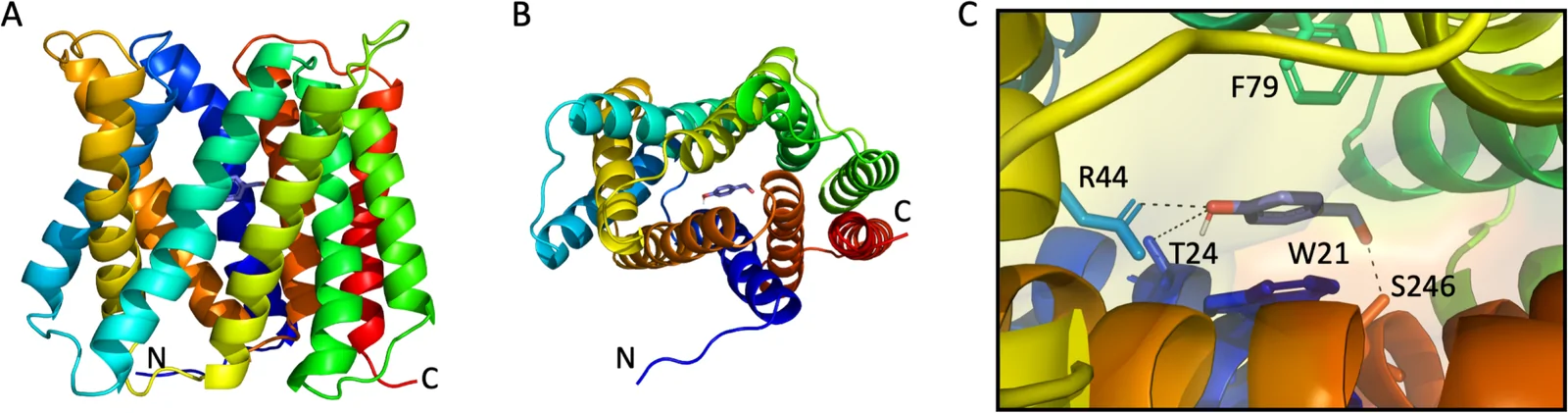
Model of PecM with 4HBA. Model created with Chai-1 and colored from blue (N-terminus) to red (C-terminus); pTM = 0.8816. (**A**) Side view with N- and C-termini at the bottom. (**B**) View with N- and C-termini toward the front. (**C**) Predicted 4HBA binding pocket. Surrounding residues are identified, with predicted stacking interactions to W21 (~3.6 Å distance) and edge-to-face interactions with F79 (~3.7 Å distance). Figure generated with PyMol 3.1.6.1.

PecM is predicted to contain two EamA domains (IPR000620), representing the ancestral five-transmembrane segment domains from which many DMT families are inferred to have evolved, including the DME family, which is thought to have arisen from an internal gene duplication event (25). Modeling of *A. fabrum* PecM using Chai-1 (26) yielded the expected structural fold consisting of 10 transmembrane helices surrounding a central cavity (Fig. 1A and B). The N- and C-termini are predicted to face the intracellular side based on the presence of positively charged residues at the N-terminus (27). The PecM model was submitted to CB-Dock2 along with the 3D conformer of 4HBA retrieved from PubChem. CB-Dock2 implements an AutoDock Vina-based molecular docking procedure to calculate the center and size of ligand-binding sites (28). The complex with the lowest Vina score (−5.3 kcal/mol) and a cavity size of 641 Å^3^ was selected for further examination (Fig. 1C). 4HBA was predicted to bind at the center of the cavity, stacking face-to-face with W21 and interacting edge-to-face with F79. Hydrogen bonding interactions to T24, R44, and S246 were also predicted. These residues are conserved among several members of the PecM protein family, including *D. dadantii* PecM (Fig. S2). The location of this binding pocket is comparable to that identified in the structure of the DMT superfamily transporter YddG from the soil bacterium *Starkeya novella*, which exports amino acids; in this structure, a central substrate-binding cavity was seen to be lined with conserved hydrophobic residues (29). The predicted PecM-4HBA complex is, therefore, consistent with a role for PecM in binding the substrate 4HBA.

**Fig 2:**
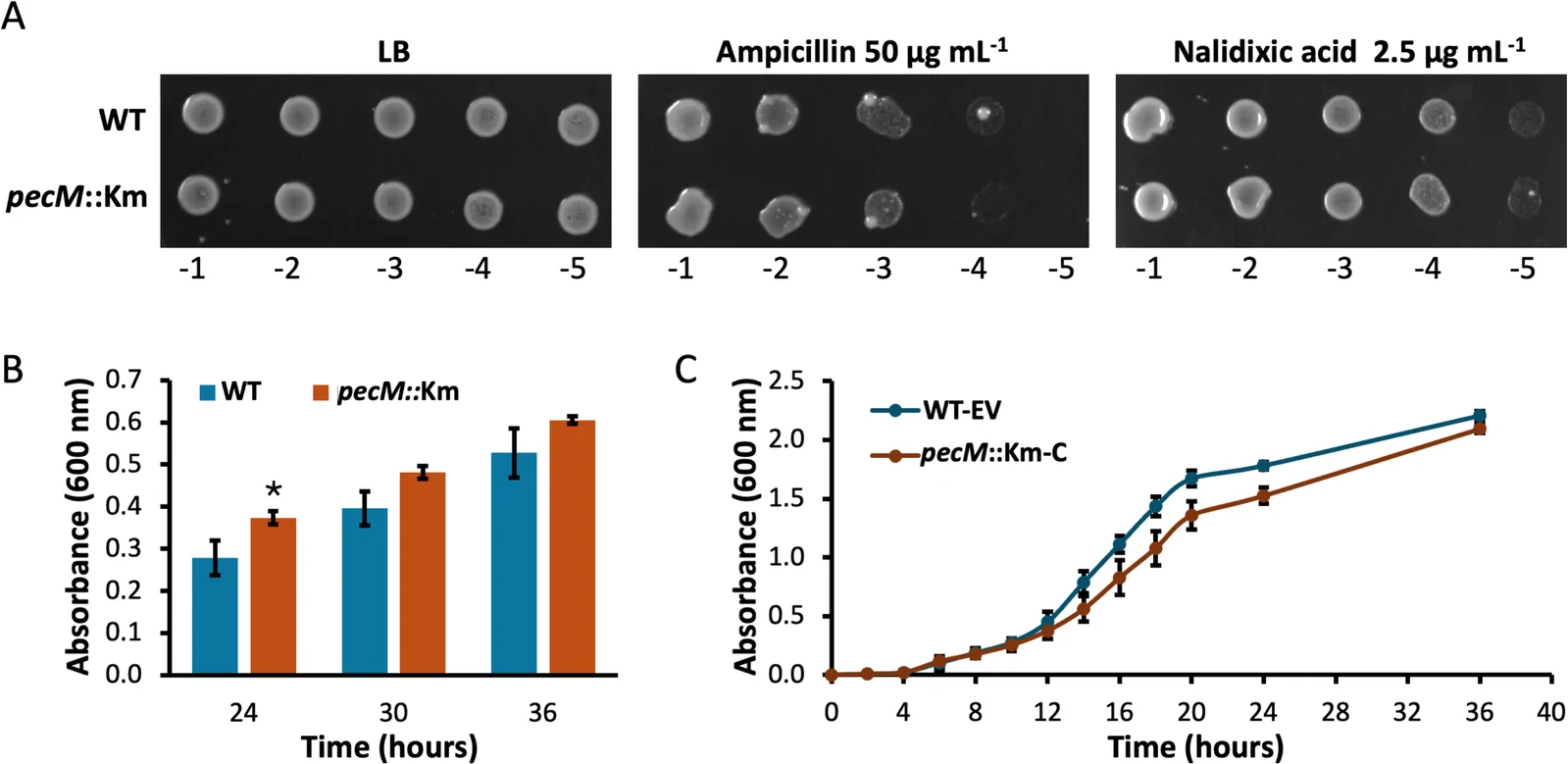
Growth and sensitivity to select antibiotics. (**A**) Exponentially growing cells were 10-fold serially diluted, and 5 μL was spotted on Luria Bertani (LB) agar plates without or with the indicated antibiotics. Images were captured after 48-h incubation at 28°C. Strains are identified at the left. Images are representative of two biological replicates. (**B**) Growth of WT and *pecM*::Km cells in AB-glucose. Absorbances were recorded at the indicated time points. Data are presented as the means from three biological replicates, and error bars represent SD. The asterisk indicates a statistically significant difference between strains, *P* < 0.05. For growth of *pecM*::Km in LB media, please see reference 18. (**C**) Growth of WT-EV and *pecM*::Km-C in LB supplemented with 60 μg/mL^−1^ chloramphenicol. Data represent means from three biological replicates.

Bacterial efflux pumps often extrude a wide range of compounds, and they contribute to events ranging from multiple antibiotic resistance to biofilm formation and host colonization (30). We, therefore, looked for evidence that *A. fabrum* PecM might export additional substrates (Fig. 2A; Fig. S3). However, a survey of a range of unrelated antibiotics, metal ions, or other potentially toxic compounds showed no evidence for PecM-mediated export.

### Phenotypes resulting from *pecM* disruption

Disruption of *pecM* does not affect growth in rich media (18). However, we observed that p*ecM*::Km cells in which *pecM* is disrupted had a modest growth advantage compared to WT cells in defined media (AB-glucose). Conversely, overexpression of *pecM* in the *pecM*::Km-C-complemented strain resulted in slower growth compared to WT cells harboring the empty plasmid (WT-EV), resulting in a generation time of ~4.3 h for WT-EV and 5.5 h for *pecM*::Km-C for growth in LB media with 60 µg/mL chloramphenicol (Fig. 2B and C). As reported previously, the expression of *pecM* in *pecM*::Km-C cells is much greater than that in WT cells (18); thus, the slower growth may be related to excess PecM causing toxicity.

Several phenotypes that might be impacted by the absence of an efflux pump were unaffected by *pecM* disruption. Biofilm formation by the *pecM*::Km cells was not altered compared to WT cells, as evidenced by comparable colony morphology and dye uptake on LB plates containing Congo Red and by crystal violet staining of biofilms formed in static cultures (Fig. S4A and B). We also observed no evidence for altered siderophore production or swimming/swarming motility (Fig. S4C and D).

We examined chemotactic motility of WT and *pecM*::Km cells in AB minimal media plates supplemented with 0.2% of various sugars, including glucose, cellobiose, or ribose. No significant difference in chemotaxis toward any of these sugars was observed between the strains. However, the distance migrated reflected that some sugars such as ribose were less favored as carbon sources (Fig. S5A). Production of the plant hormone and signaling molecule salicylic acid (SA) is induced upon bacterial infection to promote the plant defense against pathogens, and plants overproducing SA are more resistant to *Agrobacterium* infection (31). While SA notably was found to be a repellent, as evidenced by the bacteria preferentially migrating away from the SA as opposed to toward it, no significant difference was seen between strains (Fig. S5B and C).

**Fig 3:**
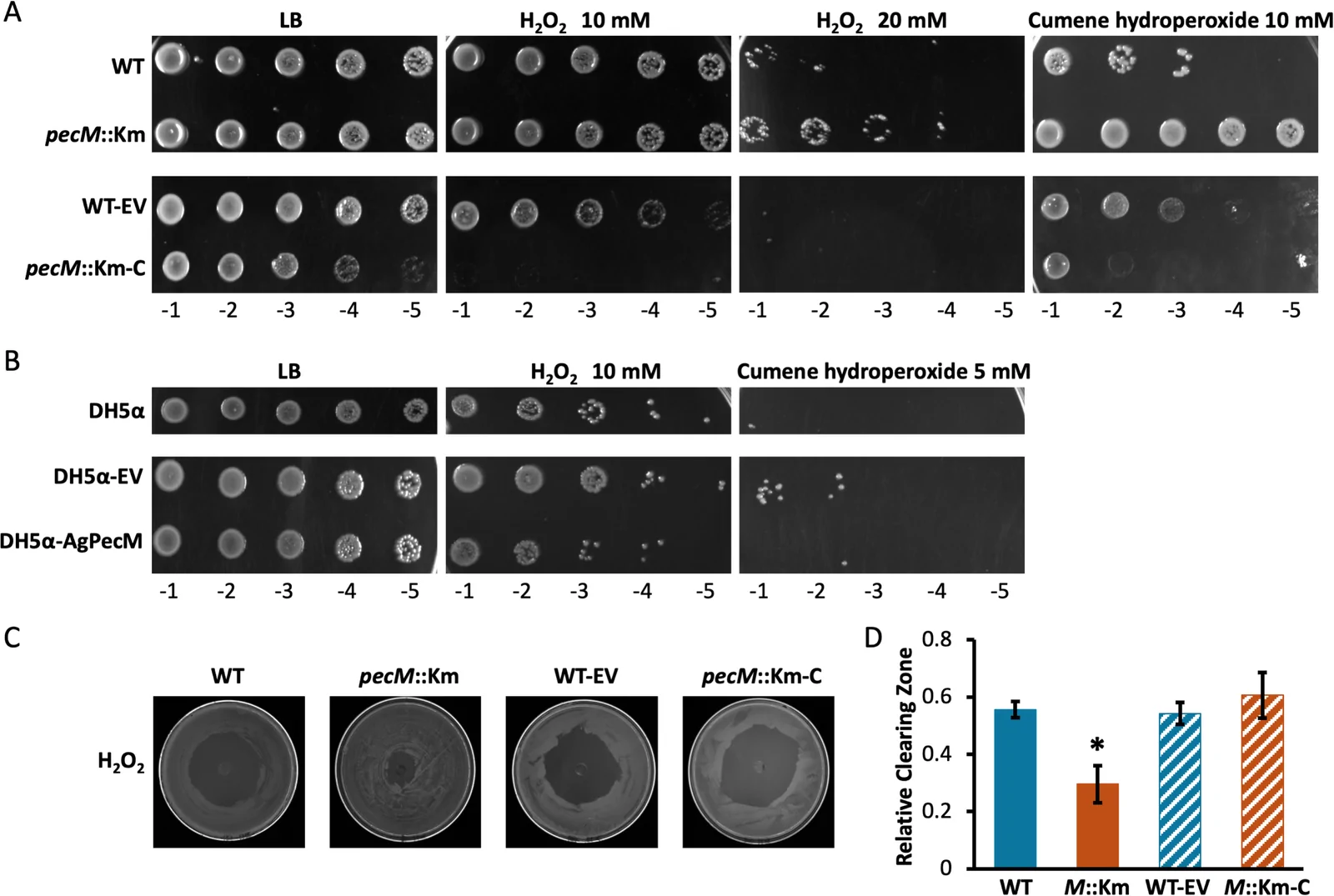
PecM is associated with sensitivity to oxidative stress. (**A**) Exponentially growing *A. fabrum* cells were exposed to the indicated oxidant for 20 min. Cells were 10-fold serially diluted (with the negative exponent of the 10-fold dilution factor provided beneath each panel), and 5 μL was spotted on LB agar plates. Images were captured after 48-h incubation at 28°C. (**B**) Exponentially growing *E. coli* cells were exposed to the indicated oxidant for 20 min. Cells were 10-fold serially diluted, and 5 μL was spotted on LB agar plates. Images were captured after 12-h incubation at 37°C. Strains are identified at the left. Images are representative of two biological replicates. (**C**) Disk diffusion assay. The WT and *pecM*::Km strains were spread on LB plates, and WT-EV and *pecM*::Km-C strains were spread on LB-Cm plates. Paper disks impregnated with 10 μL of 30% H_2_O_2_ were placed at the center, and plates were incubated at 28°C for 48 h. (**D**) Quantitation of clearing zones. Clearing zone diameters are reported relative to the diameter of the plate. The asterisk indicates a statistically significant difference relative to WT cells (*P* < 0.05) from three biological replicates.

Responses to oxidative stress were assessed by exposing exponentially growing cultures to oxidant for 20 min, followed by spotting serially diluted cultures on LB agar plates. As shown in Fig. 3A, disruption of *pecM* resulted in a marked resistance to both inorganic (H_2_O_2_) and organic (cumene hydroperoxide) oxidants. By contrast, the complemented strain *pecM*::Km-C was much more sensitive to these oxidants by comparison to WT-EV carrying the empty vector. Expression of *pecM* in *E. coli* DH5a or SM101 also conferred increased sensitivity to oxidants (Fig. 3B and data not shown). We verified the observed resistance to oxidative stress resistance of the *pecM*::Km strain by performing a disk diffusion assay in which disks impregnated with H_2_O_2_ were placed at the center of plates inoculated with the identified strains (Fig. 3C). Sensitivity of WT cells to the diffusing oxidant was evidenced by a marked clearing zone surrounding the disk, whereas *pecM*::Km cells were more resistant. Complementing the *pecM*::Km cells with *pecM* expressed from a plasmid completely restored sensitivity (Fig. 3C and D). Taken together, these analyses indicate that overt phenotypes resulting from *pecM* disruption involve oxidative stress responses.

### Physiological consequences of *pecM* disruption

For clues to the basis for the oxidative stress resistance characteristic of *pecM*::Km cells, we compared transcriptome and proteome changes imposed by disruption of PecM. Using an absolute log_2_ fold change cutoff of 1, we detected 30 differentially expressed genes (DEGs) in *pecM*::Km cells, of which six accumulated and the remainder were depleted (Table 1, Table S1). None of these DEGs provided immediate clues to the observed resistance to oxidative stress characteristic of *pecM*::Km cells (Fig. 3A). The examples of depleted mRNAs include *dmeF*, which encodes a Co(II)/Ni(II) efflux transporter; however, we did not observe altered sensitivity to these metal ions in the *pecM*::Km strain (Fig. S3B). For possible hidden clues to the oxidative stress resistance, we examined additional DEGs; RirA and MbfA have been associated with maintaining iron homeostasis (32, 33). In the *pecM*::Km cells, the corresponding mRNAs were depleted, albeit not reaching the imposed log_2_ fold change cutoff (Table S1). However, we observed no change in response to iron limitation as imposed by addition of iron chelators and no change in sensitivity to the iron-sensitive antibiotic streptonigrin as a result of *pecM* disruption (Fig. S6A). While we noted marked depletion of the mRNAs encoding lipid A-disaccharide synthase N-terminal domain-containing protein and phosphoethanolamine-lipid A transferase (Table 1), this did not correlate with altered resistance to the cationic antibiotic polymyxin E, which depends on lipid A for association (Fig. S3A).

**TABLE 1:** Examples of differentially accumulating transcripts in *pecM*::Km cells^*a*^

Gene ID new/old tag	Protein ID	Log2FC	Log2FC SE	Regulation	Protein name/function
*ATU_RS09755*/*Atu1997*	Q7CY41	1.43	0.30	Up	PepSY domain-containing protein Sulfite reductase CysJ
*ATU_RS21875*/*Atu4664*	Q7CVJ2	1.01	0.29	Up	ABC transporter ATP-binding protein (a-galactoside)
*ATU_RS04405*/*Atu0895*	A9CJS1	1.01	0.29	Up	ABC transporter substrate-binding protein (glycine-betaine)
*ATU_RS22610*/*Atu4816*	A9CH63	−2.74	0.70	Down	Glycosyltransferase family 4 protein
*ATU_RS10835*/*Atu2221*	A9CI58	−2.05	0.45	Down	Lipid-A-disaccharide synthase N-terminal domain-containing protein
*ATU_RS22370*/*Atu4766*	A9CH40	−1.99	0.38	Down	Hypothetical protein
*ATU_RS18770*/*Atu4020*	A9CFY6	−1.74	0.32	Down	Phosphoethanolamine–lipid A transferase
*ATU_RS10840*/*Atu2222*	Q7CXL0	−1.56	0.28	Down	Glycosyltransferase family 2 protein
*ATU_RS04150*/*Atu0843*	Q7D0J8	−1.48	0.33	Down	Heavy metal translocating P-type ATPase
*ATU_RS24285*/*Atu5069*	A9CLQ6	−1.46	0.28	Down	ABC transporter permease (peptide/nickel)
*ATU_RS22375*/*Atu4767*	Q7CVT6	−1.45	0.36	Down	Curli-like amyloid fiber formation chaperone CsgH
*ATU_RS04385*/*Atu0891*	Q7D0F5	−1.40	0.28	Down	CDF family Co(II)/Ni(II) efflux transporter DmeF
*ATU_RS04020*/*Atu0817*	Q7D0L8	−1.22	0.32	Down	Sulfate adenylyltransferase subunit CysD

^*a*^ Log2FC, log2 fold change; SE, standard error.

**Fig 4:**
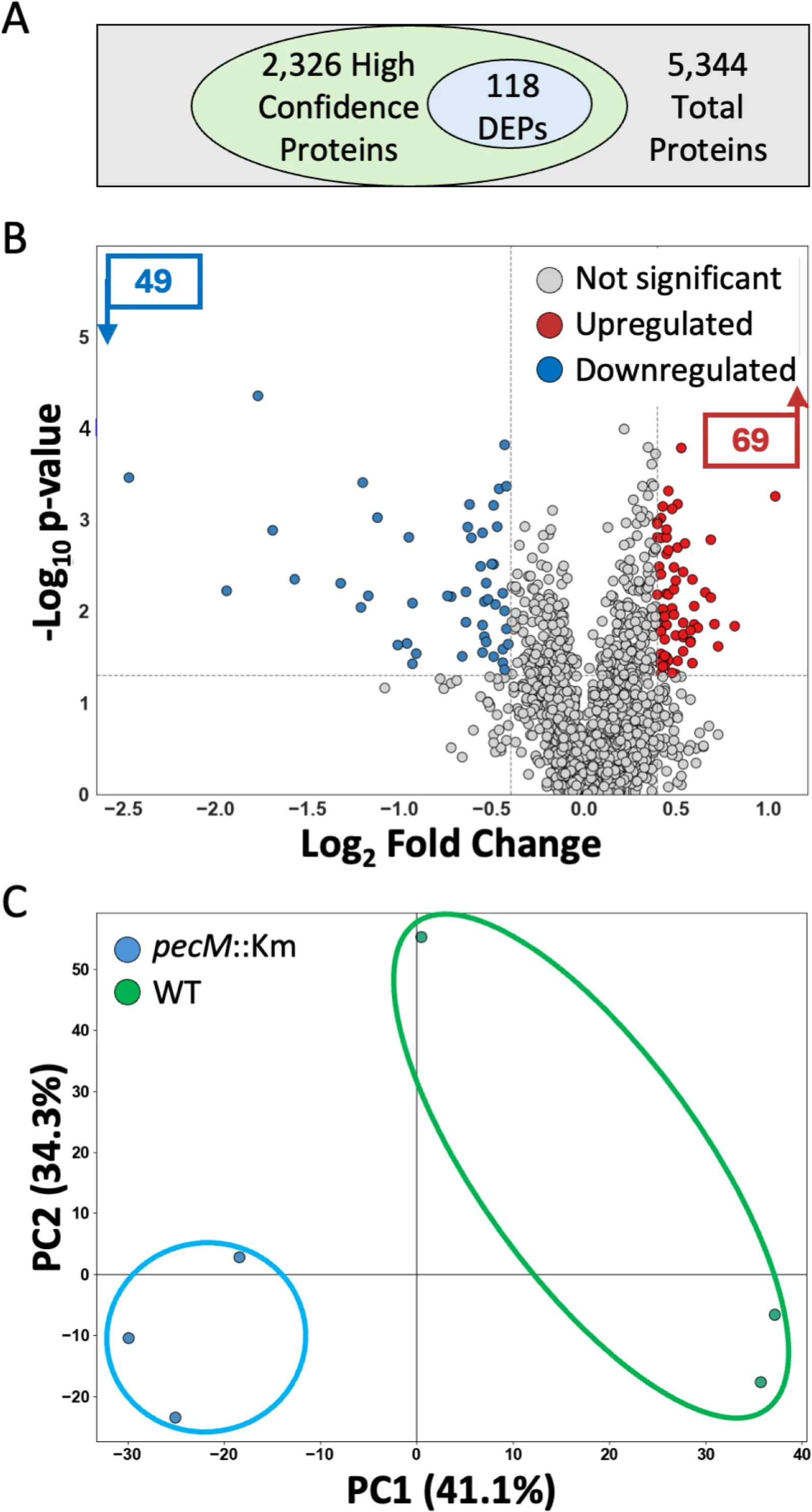
Proteome remodeling in the *pecM*::Km strain. (**A**) Differentially expressed proteins (DEPs) in *pecM*::Km cells relative to the full proteome. Among 2,326 proteins confidently identified, 118 were differentially expressed relative to WT (|log_2_ fold change| ≥ 0.4; *P* < 0.05). (**B**) Volcano plot visualizing differential protein accumulation. Proteins meeting both log_2_ fold change and significance thresholds are highlighted in red (69 upregulated) and blue (49 downregulated). (**C**) PCA of proteomic profiles. Each point represents a biological replicate, with colors representing WT (green) and *pecM*::Km cells (blue).

By comparison, 118 proteins differentially accumulated in *pecM*::Km cells based on an absolute log_2_ fold change of 0.4 and *P* < 0.05. Of 5,344 proteins indexed in UniProt, 2,326 were detected with high confidence. Among these, 69 accumulated while 49 were depleted (Fig. 4; Table 2; Tables S2 and S3, which also include information about parameters such as database search scores and statistical significance). The principal component analysis (PCA) revealed especially tight clustering for *pecM*::Km cells (Fig. 4C).

**TABLE 2:** Examples of differentially accumulating proteins in *pecM*::Km cells^*a*^

Protein ID	Gene ID	Log2FC	Log2FC SE	Regulation	Protein name/function
Q8UBZ8	*rimM*	0.69	0.08	Up	Ribosome maturation factor RimM
A9CET3	*Atu3174*	0.66	0.06	Up	ABC transporter, substrate-binding protein (sugar)
A9CJ57	*Atu1388*	0.62	0.08	Up	ABC transporter, substrate-binding protein (amino acid)
Q8U549	*Atu2283*	0.59	0.07	Up	Pseudoazurin
Q8UJ52	*rbfA*	0.53	0.01	Up	Ribosome-binding factor A
A9CJW8	*Atu0780*	0.49	0.07	Up	Thiol:disulfide interchange protein DsbD
Q8UG34	*azoR*	0.45	0.08	Up	FMN-dependent NADH:quinone oxidoreductase
A9CJ52	*Atu1394*	0.42	0.09	Up	Glutathione S-transferase
A9CIL6	*gloA*	0.42	0.10	Up	Lactoylglutathione lyase
A9CJW9	*Atu0779*	0.40	0.05	Up	Glutathione-dependent peroxiredoxin
Q8U9T8	*rpsU3*	−2.47	0.13	Down	Small ribosomal subunit protein bS21C
Q7CU65	*gshB*	−1.94	0.17	Down	Glutathione synthetase
A9CLR9	*Atu5045*	−1.77	0.06	Down	Transcriptional regulator, AbiEi antitoxin, Type IV TA system
Q7D046	*Atu1028*	−1.69	0.10	Down	NYN domain-containing protein
Q8UEQ0	*tatB*	−1.32	0.13	Down	Sec-independent protein translocase protein TatB
A9CIX5	*yajC*	−0.72	0.05	Down	Sec translocon accessory complex subunit YajC
Q7CSN8	*ppiD*	−0.55	0.07	Down	Parvulin-like PPIase
Q7CY03	*Atu2043*	−0.54	0.04	Down	Probable periplasmic serine endoprotease DegP-like
Q8UH15	*tam*	−0.50	0.06	Down	Trans-aconitate 2-methyltransferase
Q8UFZ7	*rplM*	−0.44	0.07	Down	Large ribosomal subunit protein uL13

^*a*^ Log2FC, log2 fold change; SE, standard error.

**Fig 5:**
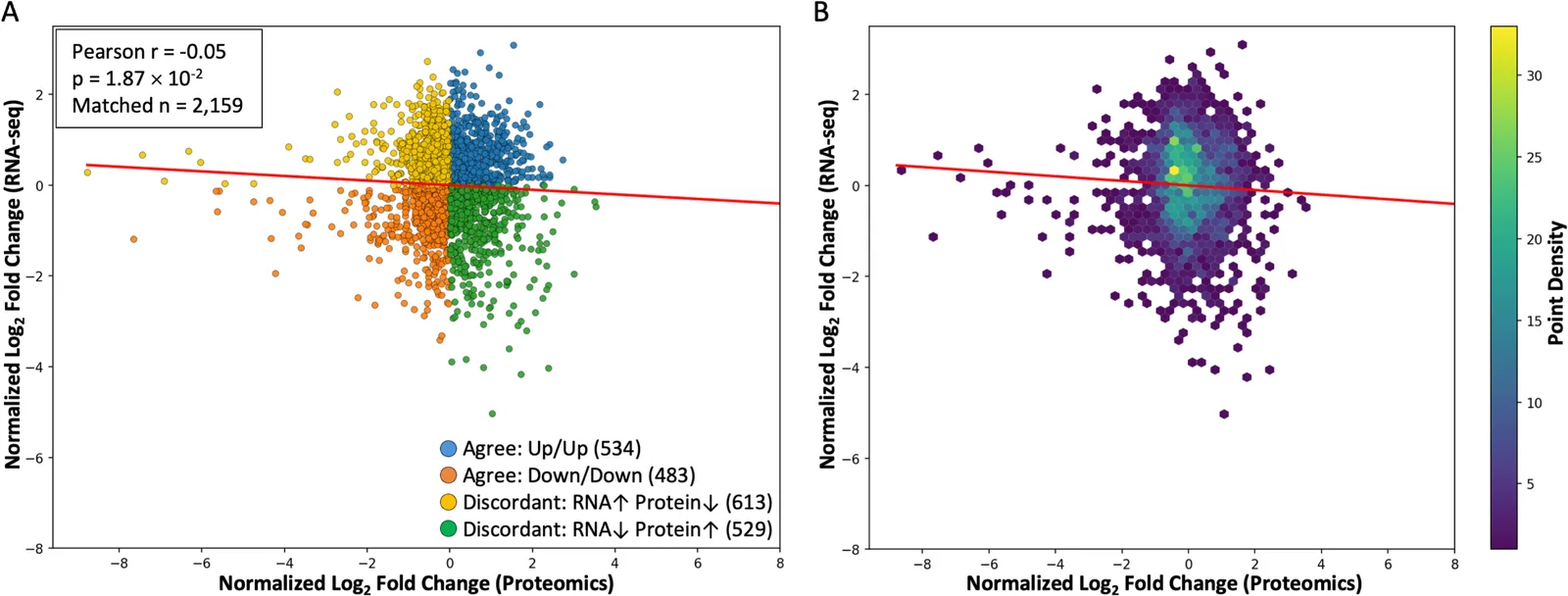
Correlation between differentially expressed mRNAs and proteins in the *pecM*::Km strain. (**A**) Scatter plot illustrating the correlation between changes in mRNA accumulation and differentially expressed proteins following z-score normalization of log_2_ fold changes. Each point represents a gene-protein pair (*n* = 2,159) detected in both data sets. The *x*-axis denotes the normalized log_2_ fold change in protein abundance, and the y-axis indicates the normalized log_2_ fold change in mRNA accumulation. The red linear regression line indicates the global trend (*r*^2^ = 0.003, indicating that only 0.3% of the variance can be attributed to a linear relationship). Colors indicate agreement between changes in mRNA and protein levels (blue; *n* = 534 and orange; *n* = 483) or lack thereof (yellow; *n* = 613 and green; *n* = 529). (**B**) Hexbin plot. Each hexagon represents multiple data points on a log scale, with color intensity indicating the density of gene-protein pairs (from yellow = high density to dark blue = low density).

The recorded changes in mRNA accumulation were integrated with the corresponding changes in protein expression. A comparison of normalized log_2_ fold changes of all confidently detected proteins and the corresponding mRNAs reflected a notable lack of a global linear correlation, with a Pearson correlation coefficient *r* = −0.05 (Fig. 5A). Similarly, the Spearman correlation coefficient of ρ = −0.06 (*P* = 0.010) reflected the effective absence of a linear relationship between DEPs and DEGs. By comparison, a hexbin plot in which each hexagon represents multiple data points on a log scale, with color intensity indicating the density of gene-protein pairs, revealed a higher density near 0 and lower density away from 0 (Fig. 5B). These plots suggest marked transcript-protein coupling for many genes, as reflected in either upregulation of both RNA and protein (Fig. 5A, blue) or downregulation of both RNA and protein (Fig. 5A, orange). However, the near-zero global correlation reflects that negative correlations also exist (Fig. 5A, yellow and green). As a result, positive and negative correlations cancel each other when calculating the global correlation. A kernel density plot of the corresponding changes primarily reflected that alterations at the mRNA levels were of modestly lower amplitude compared to proteome changes (Fig. S7).

**Fig 6:**
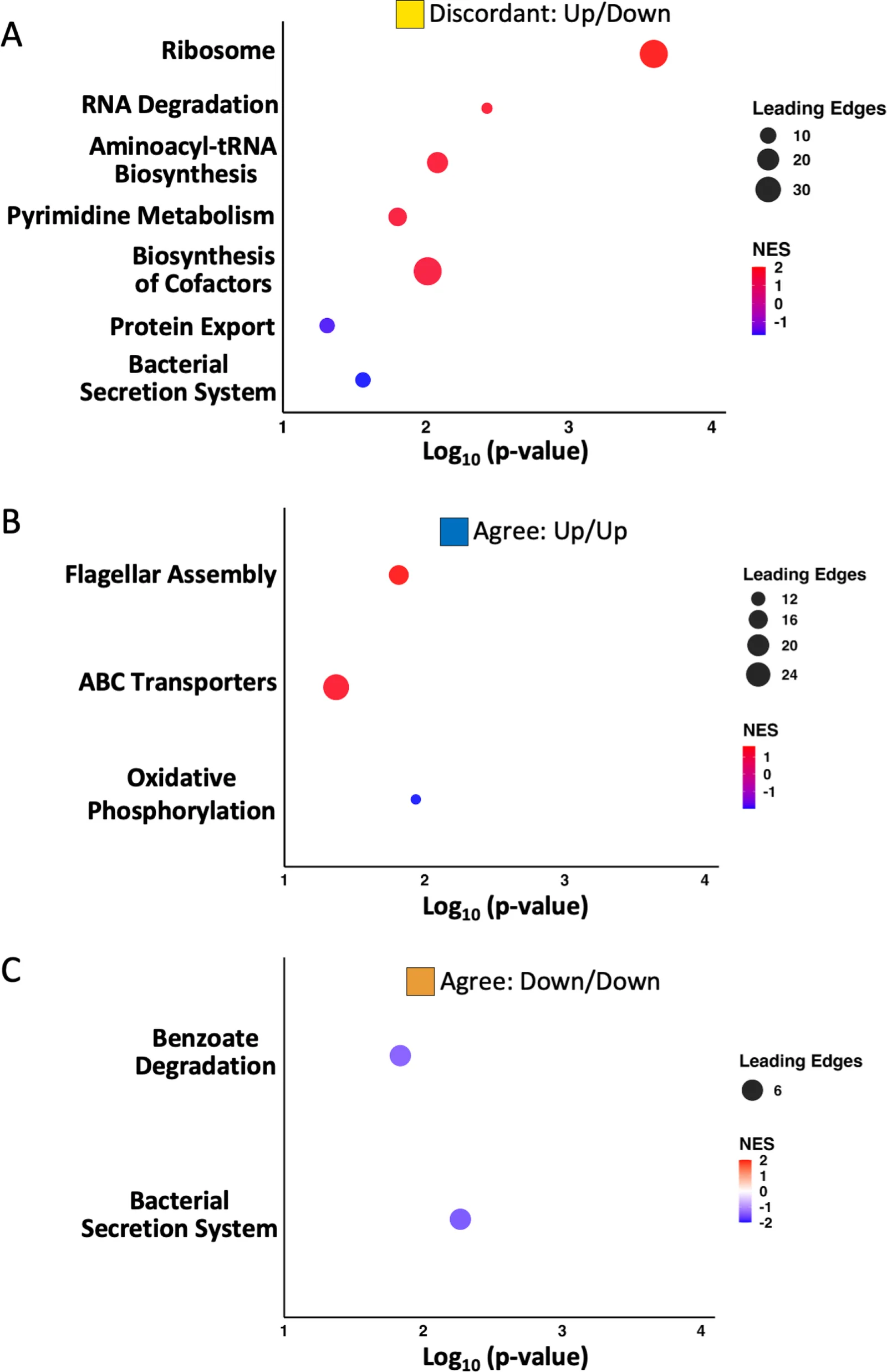
KEGG pathway enrichment for integrated transcriptomic and proteomic responses. Bubble plots show GSEA results based on a combined ranking of RNA-seq and proteomic *z*-scores, where the *x*-axis represents −log₁₀(*P*-value), the *y*-axis lists pathways, bubble size reflects the number of leading-edges, and color indicates the normalized enrichment score (NES), with red representing positive enrichment and blue representing negative enrichment. (**A)** Increased transcript levels and decreased protein abundance (yellow in Fig. 5A). (**B**) Increases in both transcript and protein levels (blue in Fig. 5A). (**C**) Decreases in both transcript and protein levels (orange in Fig. 5A). No significant pathways were identified for genes with decreased transcript levels and increased protein abundance (green in Fig. 5A).

To identify if DEG/DEP pairs for which a correlation—or lack thereof—existed was associated with specific cellular pathways, we implemented a gene set enrichment analysis (GSEA) followed by mapping to the Kyoto Encyclopedia of Genes and Genomes (KEGG) pathways. This analysis was performed separately for DEG/DEP pairs corresponding to each of the four quadrants in Fig. 5A (Fig. 6; Tables S4 to S7). The advantage of GSEA is that it can reveal the effect of individually modest, but collectively substantial changes (34). Therefore, the full ranked list of proteins was used, without imposing the bias or information loss that would be associated with implementation of an arbitrary fold change or significance cutoff. This analysis revealed that several pathways mapped to the category characterized by increased mRNA accumulation but decreased protein accumulation (Fig. 6A; yellow in Fig. 5A), and a few pathways mapped to the categories for which both mRNA and protein levels changed concordantly (Fig. 6B and C; blue and orange in Fig. 5A). This indicates that while no global correlation was observed between changes in mRNA and protein levels on disruption of *pecM*, concordance or discordance between DEGs and DEPs was not completely random.

The small ribosomal protein bS21C was markedly depleted in *pecM*::Km cells (Table 2). This protein enters the 30S small ribosomal subunit late in the assembly process, and it is important for base pairing between the anti-Shine-Dalgarno sequence in the 16S rRNA and the Shine-Dalgarno sequence in the mRNA (35). *A. fabrum* encodes three bS21 paralogs, a phenomenon also characteristic of *Francisella tularensis*, for which incorporation of a specific bS21 paralog as a constituent of a translating ribosome affects the abundance of a subset of proteins (36). We, therefore, speculate that differential accumulation of bS21 paralogs may result in ribosome heterogeneity and that the *A. fabrum* bS21 paralogs may likewise impose on the ribosome a preference for translating a specific subset of mRNAs, resulting in differential accumulation of proteins and contributing to the lack of global correlation between DEGs and DEPs. Changes to ribosome composition or function are also supported by the observation that the ribosome maturation factor RimM and ribosome binding factor A (RbfA) accumulate (Table 2).

We have previously reported that 4HBA is a substrate for PecM, as, for instance, evidenced by accumulation of this compound in *pecM*::Km cells (18). As an aromatic aldehyde, 4HBA is a chemically reactive electrophile that can react with cellular nucleophiles such as thiol groups on proteins and glutathione, leading to disruption of redox homeostasis. In addition, 4HBA may undergo spontaneous or enzymatic oxidation of the hydroxyl group in the presence of oxygen and trace metal ions such as Fe^2+^ to generate a semiquinone, a process that produces superoxide anion and H_2_O_2_. Semiquinones are highly reactive and can generate further reactive oxygen species (ROS) in the presence of oxygen, exacerbating the induction of oxidative stress (37). In support of such 4HBA-induced redox events, we note that *pecM*::Km cells exhibit several tell-tale signs of oxidative stress, such as accumulation of glutathione S-transferase and glutathione-dependent peroxiredoxin (Table 2), both of which protect from oxidative stress. In addition, *pecM*::Km cells accumulate the thiol:disulfide interchange protein DsbD, which is a membrane-bound protein that plays a critical role in maintaining redox homeostasis in the periplasm by transferring electrons from the cytoplasm to the periplasm, thus enabling reductive disulfide bond isomerization and repair. Pseudoazurin also accumulates; this periplasmic protein functions in electron transfer, delivering electrons to terminal oxidases involved in oxygen or nitrogen respiration. Taken together, upregulation of all these proteins suggests that the cells are experiencing oxidative or electrophile stress. Consistent with accumulation of these proteins, the *pecM* disrupted strain was more resistant to oxidative stress (Fig. 3).

Rapid detoxification of 4HBA by oxidizing it to the corresponding carboxylic acid is critical to lessening the aforementioned redox complications, and it likely requires NAD^+^ as a cofactor, thereby generating NADH and altering the NADH:NAD^+^ ratio. We note here the upregulation of FMN-dependent NADH:quinone oxidoreductase (NQO; Q8UG34), which converts NADH to NAD^+^ (Table 2). We, therefore, speculate that upregulation of NQO occurs because the cells need to direct electrons from NADH into the quinone pool. The protein family to which this NQO belongs has been shown in other bacterial species to play a role in oxidative defense (38).

One possible consequence of an altered NADH:NAD^+^ ratio could be reduced flux through glycolysis and a buildup of upstream intermediates that might lead to overflow into side reactions, such as the conversion of glucose or fructose (and their 6-phosphates) to the corresponding hexose alcohols, sorbitol or mannitol. In support of this interpretation, we note that an (unknown) hexose alcohol accumulates (3.8-fold) in *pecM*::Km cells (18). Considering that sorbitol may be the most likely hexose alcohol to accumulate, we tested the growth of *pecM*::Km cells with sorbitol as the sole carbon source. No difference in growth compared to WT cells was observed, suggesting that if sorbitol accumulates in *pecM*::Km cells, it is for reasons other than deficient catabolism (Fig. S6B).

Reduced flux through the citric acid cycle may also occur when PecM is missing. As reported previously, degradation of 4HBA leads to formation of succinate, which also accumulates (2.6-fold) in *pecM*::Km cells (18). Its accumulation indicates that flux through the citric acid cycle is impaired or downregulated, perhaps due to reduced activity of succinate dehydrogenase whose iron-sulfur clusters are sensitive to ROS-mediated damage. Aconitase, which converts citrate to isocitrate via *cis*-aconitate, is also prone to oxidative damage. Under such conditions, *trans*-aconitate is generated, and this compound acts as an inhibitor of aconitase (39). The enzyme *trans*-aconitate methyltransferase detoxifies *trans*-aconitate; this enzyme is downregulated in *pecM*::Km cells, perhaps as a reflection of reduced aconitase activity (Table 2). Consistent with reduced flux through the citric acid cycle in *pecM*::Km cells (18), we note that the accumulation of glutamate that is characteristic of *pecM*::Km cells is reversed in the complemented strain (Fig. S6C).

A remarkable effect of the *pecM* disruption is the upregulation of a large number of substrate-binding components of ABC transporters (Table 2 and Table S2). Considering the effects on redox state and central metabolism that are likely induced by accumulation of 4HBA, such upregulation may serve several functions. One might be to scavenge for alternate nutrients or more readily metabolizable substrates, as evidenced by transporters with specificity for ribose or amino acids. We also note upregulation of transporters expected to import redox-active cofactors such as molybdate and iron and the osmoprotectant glycine betaine, which may aid in restoration of homeostasis.

When *pecM*::Km cells are grown in defined media, a modest growth advantage is observed (Fig. 2B). In this scenario, the burden of 4HBA accumulation would presumably be low. As reported previously, *pecM* is significantly induced by 4HBA (but not by 4-hydroxybenzoate) (18), indicating that the expression of PecM is closely linked to the presence of 4HBA. The modest increase in growth in defined media, therefore, reflects a condition-specific outcome, and it is not an indication that PecM universally reduces fitness. Rather, the expectation would be for PecM to be advantageous under conditions when 4HBA accumulates. For instance, 4HBA may be encountered in the rhizosphere as a component of plant exudates (21, 23), conditions under which PecM would then be expected to confer a fitness advantage. The observed phenotypes of the *pecM*::Km strain likely reflect a physiological trade-off that is specific to the experimental conditions. Disruption of *pecM* results in intracellular accumulation of 4HBA and in induction of oxidative/electrophile stress-associated proteins, at least during growth in rich medium where absence of PecM does not confer a growth advantage (18). This is consistent with the absence of PecM producing a pre-adapted state that increases resistance to subsequent oxidative challenge but is accompanied by a relative reduction in the growth rate as compared to growth in defined medium. Such trade-offs between growth and stress resistance are well documented (40).

Taken together, our data indicate that absence of PecM results in widespread metabolic changes, including the accumulation of proteins associated with oxidative stress responses and cellular protection pathways, changes that likely result from the oxidative or electrophile stress imposed by 4HBA accumulation. We, therefore, infer that PecM serves as a critical metabolic relief valve to maintain cellular homeostasis.

More broadly, our findings add to growing evidence that bacterial efflux pumps should not be viewed solely as detoxification systems but also as integral components of cellular physiology. The observation that loss of PecM remodels the proteome and enhances oxidative stress resistance suggests that intracellular retention of metabolic intermediates can remodel stress-response pathways. Our data, therefore, support a model in which impaired export of an endogenous metabolite leads to widespread metabolic and physiological remodeling, linking metabolite transport to stress adaptation, and they suggest that endogenous metabolite accumulation may represent an underappreciated mechanism by which transport systems influence bacterial physiology.

## MATERIALS AND METHODS

### Bacterial strains and growth conditions

The disarmed strain *A. fabrum* GV3101 pMP90, referred to as WT, is a derivative of the C58 strain in which the T-DNA is deleted from the nopaline-type Ti plasmid (pTiC58). The *pecM::*Km strain in which *pecM* is disrupted, WT-EV (WT strain carrying empty plasmid pBBRBAD2), *pecM::*Km strain carrying empty pBBRBAD2 (*pecM::*Km-EV), and the *pecM::*Km complemented strain (*pecM*::Km-C) were constructed as described (18). The WT and *pecM::*Km cells were grown in LB media, whereas WT-EV, *pecM::*Km-EV, and *pecM*::Km-C were grown in LB-Cm (LB with 60 µg/mL chloramphenicol). Cultures were grown at 28°C with shaking. For some assays, AB medium (*Agrobacterium* minimal medium) supplemented with 0.2% glucose as a carbon source (AB-glucose) was used; AB is a modified M9 minimal medium (41).

*E. coli* DH5α was grown in LB, while DH5α-EV and DH5α-AgPecM were grown in LB-Cm. DH5α-EV carries empty pBBRBAD2. DH5α-AgPecM carries plasmid harboring *A. fabrum* PecM in pBBRBAD2 (18). *E. coli* strains were grown at 37°C with shaking.

### Sensitivity assays

Plate assays were used to assess sensitivity to miscellaneous compounds. *A. fabrum* cultures grown ~48 h at 28°C in LB were diluted 1:100 in fresh media with or without Cm as specified above and grown to OD_600_ ≈0.6. Five microliters of 10-fold serially diluted culture was spotted on LB agar plates or AB-glucose agar plates containing the indicated concentration of the identified compounds. The compounds included in these assays were as follows: ampicillin, bipyridyl, chloramphenicol, ethidium bromide, nalidixic acid, novobiocin, polymyxin E, streptonigrin, and tetracycline and the salts NaCl, CdCl_2_, CoCl_2_, MgCl_2_, and NiCl_2_. After incubation at 28°C for at least 48 h, images were captured. Assays with *E. coli* strains exposed to H_2_O_2_ or cumene hydroperoxide were performed similarly, except that cultures were grown at 37°C and plates were incubated for 12 h.

For sensitivity to oxidants, cells were treated with the indicated concentrations of cumene hydroperoxide or hydrogen peroxide for 20 min with shaking after cultures reached an OD_600_ ≈0.6, followed by spotting of 10-fold serial dilutions on LB agar plates. The assays were repeated at least twice.

For disk diffusion assays, WT and *pecM*::Km strains were grown at 28°C in LB with aeration, while WT-EV and *pecM*::Km-C strains were grown in LB-Cm. Cells were harvested at an OD_600_ ≈1.0, and 100 μL of the culture was spread on LB or LB-Cm plates, as appropriate. Paper disks impregnated with 10 μL of 30% H_2_O_2_ were placed at the center, and the plates were incubated at 28°C for 2 days. The clear zone of inhibition (including the disk) was determined for each plate as the average of perpendicular diameters and reported relative to the diameter of the plate. The data represent three biological replicates, and they are reported as mean ± SD. Statistical significance was determined based on Student’s *t*-test.

### RNA sequencing

Three biological replicates of wild-type and *pecM*::Km cells were used. Single colonies were inoculated in 3 mL LB media and grown at 28°C for 48 h with shaking, and these cultures were used to inoculate fresh LB at a 1:100 dilution. Cells from 3 mL culture were collected by centrifugation at 16,000 × *g* for 2 min when cultures reached OD_600_ ≈1.0. The cell pellets were washed twice with diethyl pyrocarbonate-treated water and stored at −80°C. RNA extraction was conducted using the Monarch Total RNA Miniprep Kit (New England BioLabs; Ipswich, MA) according to the manufacturer’s instructions. The integrity of the extracted RNA was assessed by agarose gel electrophoresis, and RNA was quantified using a NanoDrop 2000c spectrophotometer. An Agilent 2100 Bioanalyzer was used to obtain the RNA Integrity Number. Library preparation and high-throughput sequencing was performed by Novogene (https://www.novogene.com). The raw reads were processed and aligned to the indexed genome, and differential gene expression between the wild-type and *pecM*::Km was identified using DESeq2 (42). Genes were considered differentially expressed if they exhibited a log_2_ fold change of ≥1 or ≤−1 and if they met the statistical significance threshold with a false discovery rate (FDR) of <0.05. For details on sequencing and bioinformatics analyses, see Supporting Information.

### Quantitative mass spectrometry proteomics

Cells were grown in LB and collected at OD_600_ ≈1.0 by centrifugation. Cells from ~33 mL of culture were harvested from the same cultures used to collect cells for RNA sequencing. Sample processing was achieved using the S-Trap method, and TMTsixplex labeling was conducted as previously described (43). After labeling, the six TMT-labeled peptide samples (three representing wild-type and three representing *pecM*::Km) were pooled and fractionated into six fractions. These six fractions were dried and resuspended in 10 µL 0.1% formic acid for injection. Mass spectrometry was performed using a Q-Exactive orbitrap mass spectrometer (Thermo Scientific, Waltham, MA) coupled to a Vanquish Neo liquid chromatography system (Thermo Scientific), and it was operated in the data-dependent acquisition mode.

Raw mass spectra were subjected to data processing using Proteome Discoverer 2.4, SP1 (PD) (44). Peak picking, spectral alignment, and peptide identification were achieved using the SEQUEST HT and MASCOT (Matrix Science, London, UK) search engines. Percolator was used to calculate FDRs at the peptide levels, while the native Proteome Discoverer protein FDR validator was used to calculate FDRs at the protein level. Peptides and proteins with an FDR <0.01 were used and only proteins for which at least two peptides were identified, of which one was unique, were included. The protein ratio calculation was set to “protein abundance based,” and the hypothesis test for *P*-value generation was set to analysis of variance (individual proteins). Multiple hypothesis correction was applied at the protein level using the Benjamini-Hochberg procedure, as implemented in PD, to control for the false discovery rate. Proteins were considered significantly differentially expressed based on log_2_ fold change ≥|0.4| and a *P*-value <0.05. The absolute log_2_ fold change cutoff of 0.4 (~1.3-fold) was chosen based on the known characteristics of isobaric labeling-based quantitative proteomics, where ratio compression caused by co-isolation and co-fragmentation reduces the apparent magnitude of measured fold changes (45, 46). This threshold exceeds the observed technical variability across replicates while maintaining statistical significance and biological relevance. For further details on sample preparation, TMT labeling, data acquisition, and data analyses, see Supporting Information.

### KEGG pathway changes

GSEA was performed to identify significantly regulated pathways using the KEGG database (47). Pathways were identified using the fgsea package in R using the full ranked list of proteins or mRNAs as the input file (48). Pathway enrichment results were visualized using custom scripts to generate bubble plots.

### Integration of RNA-seq and proteomics data

The log_2_-transformed transcript and protein fold changes were matched by locus tag, retaining features quantified in both data sets (*n* = 2,159 pairs), with both values expressed relative to the wild-type strain. The global relationship between transcript and protein fold changes was evaluated using both Pearson (linear) and Spearman (rank-order) correlation coefficients, calculated with the pearsonr and spearmanr functions of the SciPy library, respectively (49). Visualization was performed using the seaborn and matplotlib libraries (50, 51). Detailed information on statistical methods was reported previously (43).

## Data Availability

The mass spectrometry proteomics data have been deposited to the ProteomeXchange Consortium via the PRIDE (52) partner repository with the data set identifier PXD069909 and 10.6019/PXD069909. The RNA-seq data have been deposited to NCBI’s Gene Expression Omnibus (53) and are accessible through GEO Series accession number GSE328778.

## SUPPLEMENTAL MATERIAL

Supplemental material msphere.00601-26-s0001.pdf Supplemental figures and expanded methods.

Captions msphere.00601-26-s0002.pdf Supplemental table captions.

Table S1 msphere.00601-26-s0003.xlsx DEGs.

Table S2 msphere.00601-26-s0004.xlsx Proteins detected by proteomics.

Table S3 msphere.00601-26-s0005.xlsb Peptides detected by proteomics.

Table S4 msphere.00601-26-s0006.xlsx Significantly enriched KEGG pathways from GSEA for pairs of accumulating DEGs and DEPs.

Table S5 msphere.00601-26-s0007.xlsx Significantly enriched KEGG pathways from GSEA for pairs of depleted DEGs and DEPs.

Table S6 msphere.00601-26-s0008.xlsx Significantly enriched KEGG pathways from GSEA for pairs of DEGs and DEPs in which the DEGs are depleted and the DEPs accumulate.

Table S7 msphere.00601-26-s0009.xlsx Significantly enriched KEGG pathways from GSEA for pairs of DEGs and DEPs in which the DEGs accumulate and the DEPs are depleted.

ASM does not own the copyrights to Supplemental Material that may be linked to, or accessed through, an article. The authors have granted ASM a non-exclusive, world-wide license to publish the Supplemental Material files. Please contact the corresponding author directly for reuse.

## References

[1] Bouwmeester H, Dong L, Wippel K, Hofland T, Smilde A. 2025. The chemical interaction between plants and the rhizosphere microbiome. Trends Plant Sci 30:1002–1019. doi:10.1016/j.tplants.2025.06.001 PMC12415530 40603217

[2] Kumawat M, Nabi B, Daswani M, Viquar I, Pal N, Sharma P, Tiwari S, Sarma DK, Shubham S, Kumar M. 2023. Role of bacterial efflux pump proteins in antibiotic resistance across microbial species. Microb Pathog 181:106182. doi:10.1016/j.micpath.2023.106182 37263448

[3] Van Dyk TK, Templeton LJ, Cantera KA, Sharpe PL, Sariaslani FS. 2004. Characterization of the *Escherichia coli* AaeAB efflux pump: a metabolic relief valve? J Bacteriol 186:7196–7204. doi:10.1128/JB.186.21.7196-7204.2004 PMC523213 15489430

[4] Whittle EE, Orababa O, Osgerby A, Siasat P, Element SJ, Blair JMA, Overton TW. 2024. Efflux pumps mediate changes to fundamental bacterial physiology via membrane potential. mBio 15:e02370-24. doi:10.1128/mbio.02370-24 PMC11481890 39248573

[5] Gelvin SB. 2017. Integration of *Agrobacterium* T-DNA into the Plant Genome. Annu Rev Genet 51:195–217. doi:10.1146/annurev-genet-120215-035320 28853920

[6] Gordon JE, Christie PJ. 2014. The *Agrobacterium* ti plasmids. Microbiol Spectr 2. doi:10.1128/microbiolspec.plas-0010-2013 PMC4292801 25593788

[7] Nwokocha GC, Adhikari P, Iqbal A, Elkholy H, Doerrler WT, Larkin JC, Grove A. 2023. Transcription factor PecS mediates *Agrobacterium fabrum* fitness and survival. J Bacteriol 205:e00478-22. doi:10.1128/jb.00478-22 PMC10367594 37314346

[8] Deochand DK, Grove A. 2017. MarR family transcription factors: dynamic variations on a common scaffold. Crit Rev Biochem Mol Biol 52:595–613. doi:10.1080/10409238.2017.1344612 28670937

[9] Nwokocha GC, Ghosh A, Grove A. 2024. Regulation of bacterial virulence genes by PecS family transcription factors. J Bacteriol 206:e00302-24. doi:10.1128/jb.00302-24:e0030224 PMC11500572 39287432

[10] Perera IC, Grove A. 2010. Urate is a ligand for the transcriptional regulator PecS. J Mol Biol 402:539–551. doi:10.1016/j.jmb.2010.07.053 20678501

[11] Charkowski A, Blanco C, Condemine G, Expert D, Franza T, Hayes C, Hugouvieux-Cotte-Pattat N, López Solanilla E, Low D, Moleleki L, Pirhonen M, Pitman A, Perna N, Reverchon S, Rodríguez Palenzuela P, San Francisco M, Toth I, Tsuyumu S, van der Waals J, van der Wolf J, Van Gijsegem F, Yang C-H, Yedidia I. 2012. The role of secretion systems and small molecules in soft-rot *Enterobacteriaceae* pathogenicity. Annu Rev Phytopathol 50:425–449. doi:10.1146/annurev-phyto-081211-173013 22702350

[12] Reverchon S, Nasser W, Robert-Baudouy J. 1994. pecS: a locus controlling pectinase, cellulase and blue pigment production in *Erwinia chrysanthemi*. Mol Microbiol 11:1127–1139. doi:10.1111/j.1365-2958.1994.tb00389.x 8022282

[13] Perera IC, Grove A. 2011. MarR homologs with urate-binding signature. Protein Sci 20:621–629. doi:10.1002/pro.588 PMC3064840 21432936

[14] Deochand DK, Meariman JK, Grove A. 2016. pH-Dependent DNA distortion and repression of gene expression by *Pectobacterium atrosepticum* PecS. ACS Chem Biol 11:2049–2056. doi:10.1021/acschembio.6b00168 27213700

[15] Huang H, Mackel BJ, Grove A. 2013. Streptomyces coelicolor encodes a urate-responsive transcriptional regulator with homology to PecS from plant pathogens. J Bacteriol 195:4954–4965. doi:10.1128/JB.00854-13 PMC3807492 23995633

[16] Bhattacharyya N, Lemon TL, Grove A. 2019. A role for *Vibrio vulnificus* PecS during hypoxia. Sci Rep 9:2797. doi:10.1038/s41598-019-39095-4 PMC6391409 30808913

[17] Wang ZC, Liu CJ, Huang YJ, Wang YS, Peng HL. 2015. PecS regulates the urate-responsive expression of type 1 fimbriae in *Klebsiella pneumoniae* CG43. Microbiology (Reading) 161:2395–2409. doi:10.1099/mic.0.000185 26385366

[18] Ghosh A, Grove A. 2025. The *Agrobacterium fabrum* efflux pump PecM is produced in response to the plant exudate 4-hydroxybenzaldehyde to avoid disruption of central metabolism. J Bacteriol 207:e00150-25. doi:10.1128/jb.00150-25 PMC12288460 40511934

[19] LoPachin RM, Gavin T. 2014. Molecular mechanisms of aldehyde toxicity: a chemical perspective. Chem Res Toxicol 27:1081–1091. doi:10.1021/tx5001046 PMC4106693 24911545

[20] Singh S, Brocker C, Koppaka V, Chen Y, Jackson BC, Matsumoto A, Thompson DC, Vasiliou V. 2013. Aldehyde dehydrogenases in cellular responses to oxidative/electrophilic stress. Free Radic Biol Med 56:89–101. doi:10.1016/j.freeradbiomed.2012.11.010 PMC3631350 23195683

[21] Eisenhauer N, Lanoue A, Strecker T, Scheu S, Steinauer K, Thakur MP, Mommer L. 2017. Root biomass and exudates link plant diversity with soil bacterial and fungal biomass. Sci Rep 7:44641. doi:10.1038/srep44641 PMC5379681 28374800

[22] Vives-Peris V, de Ollas C, Gómez-Cadenas A, Pérez-Clemente RM. 2020. Root exudates: from plant to rhizosphere and beyond. Plant Cell Rep 39:3–17. doi:10.1007/s00299-019-02447-5 31346716

[23] Bhattacharya A, Sood P, Citovsky V. 2010. The roles of plant phenolics in defence and communication during *Agrobacterium* and *Rhizobium* infection. Mol Plant Pathol 11:705–719. doi:10.1111/j.1364-3703.2010.00625.x PMC6640454 20696007

[24] Jiménez JI, Miñambres B, García JL, Díaz E. 2002. Genomic analysis of the aromatic catabolic pathways from *Pseudomonas putida* KT2440. Environ Microbiol 4:824–841. doi:10.1046/j.1462-2920.2002.00370.x 12534466

[25] Jack DL, Yang NM, Saier MH Jr. 2001. The drug/metabolite transporter superfamily. Eur J Biochem 268:3620–3639. doi:10.1046/j.1432-1327.2001.02265.x 11432728

[3] Boitreaud J, Dent J, McPartlon M, Meier J, Reis V, Rogozhnikov A, Wu K, Chai Discovery. 2024. Chai-1: decoding the molecular interactions of life. bioRxiv. doi:10.1101/2024.10.10.615955

[27] vonHeijne G. 1989. Control of topology and mode of assembly of a polytopic membrane protein by positively charged residues. Nature 341:456–458. doi:10.1038/341456a0 2677744

[28] Liu Y, Yang X, Gan J, Chen S, Xiao Z-X, Cao Y. 2022. CB-Dock2: improved protein–ligand blind docking by integrating cavity detection, docking and homologous template fitting. Nucleic Acids Res 50:W159–W164. doi:10.1093/nar/gkac394 PMC9252749 35609983

[29] Tsuchiya H, Doki S, Takemoto M, Ikuta T, Higuchi T, Fukui K, Usuda Y, Tabuchi E, Nagatoishi S, Tsumoto K, Nishizawa T, Ito K, Dohmae N, Ishitani R, Nureki O. 2016. Structural basis for amino acid export by DMT superfamily transporter YddG. Nature 534:417–420. doi:10.1038/nature17991 27281193

[30] Pasqua M, Grossi M, Zennaro A, Fanelli G, Micheli G, Barras F, Colonna B, Prosseda G. 2019. The varied role of efflux pumps of the MFS family in the interplay of bacteria with animal and plant cells. Microorganisms 7:285. doi:10.3390/microorganisms7090285 PMC6780985 31443538

[31] Yuan ZC, Edlind MP, Liu P, Saenkham P, Banta LM, Wise AA, Ronzone E, Binns AN, Kerr K, Nester EW. 2007. The plant signal salicylic acid shuts down expression of the vir regulon and activates quormone-quenching genes in Agrobacterium. Proc Natl Acad Sci USA 104:11790–11795. doi:10.1073/pnas.0704866104 PMC1905925 17606909

[32] Hibbing ME, Fuqua C. 2011. Antiparallel and interlinked control of cellular iron levels by the Irr and RirA regulators of *Agrobacterium tumefaciens*. J Bacteriol 193:3461–3472. doi:10.1128/JB.00317-11 PMC3133315 21602352

[33] Bhubhanil S, Chamsing J, Sittipo P, Chaoprasid P, Sukchawalit R, Mongkolsuk S. 2014. Roles of *Agrobacterium tumefaciens* membrane-bound ferritin (MbfA) in iron transport and resistance to iron under acidic conditions. Microbiology 160:863–871. doi:10.1099/mic.0.076802-0 24600024

[34] Subramanian A, Tamayo P, Mootha VK, Mukherjee S, Ebert BL, Gillette MA, Paulovich A, Pomeroy SL, Golub TR, Lander ES, Mesirov JP. 2005. Gene set enrichment analysis: a knowledge-based approach for interpreting genome-wide expression profiles. Proc Natl Acad Sci USA 102:15545–15550. doi:10.1073/pnas.0506580102 PMC1239896 16199517

[35] Backendorf C, Ravensbergen CJC, Van der Plas J, van Boom JH, Veeneman G, Van Duin J. 1981. Basepairing potential of the 3′ terminus of 16S RNA: dependence on the functional state of the 30S subunit and the presence of protein S21. Nucl Acids Res 9:1425–1444. doi:10.1093/nar/9.6.1425 PMC326767 7232220

[36] Trautmann HS, Ramsey KM. 2022. A ribosomal protein homolog governs gene expression and virulence in a bacterial pathogen. J Bacteriol 204:e00268-22. doi:10.1128/jb.00268-22 PMC9578407 36121290

[37] Imlay JA. 2015. Diagnosing oxidative stress in bacteria: not as easy as you might think. Curr Opin Microbiol 24:124–131. doi:10.1016/j.mib.2015.01.004 PMC4380616 25666086

[38] Green LK, La Flamme AC, Ackerley DF. 2014. *Pseudomonas aeruginosa* MdaB and WrbA are water-soluble two-electron quinone oxidoreductases with the potential to defend against oxidative stress. J Microbiol 52:771–777. doi:10.1007/s12275-014-4208-8 25085734

[39] Huang H, Grove A. 2013. The transcriptional regulator TamR from *Streptomyces coelicolor* controls a key step in central metabolism during oxidative stress. Mol Microbiol 87:1151–1166. doi:10.1111/mmi.12156 23320788

[40] Ferenci T. 2016. Trade-off mechanisms shaping the diversity of bacteria. Trends Microbiol 24:209–223. doi:10.1016/j.tim.2015.11.009 26705697

[41] Clark DJ, Maaløe O. 1967. DNA replication and the division cycle in *Escherichia coli*. J Mol Biol 23:99–112. doi:10.1016/S0022-2836(67)80070-6

[42] Love MI, Huber W, Anders S. 2014. Moderated estimation of fold change and dispersion for RNA-seq data with DESeq2. Genome Biol 15:550. doi:10.1186/s13059-014-0550-8 PMC4302049 25516281

[43] Al-Tohamy A, Donnarumma F, Grove A. 2025. Transcriptome-proteome profiling in *Burkholderia thailandensis* during the transition from exponential to stationary phase. J Proteome Res 24:4082–4097. doi:10.1021/acs.jproteome.5c00223 PMC12322963 40680064

[44] Orsburn BC. 2021. Proteome discoverer-a community enhanced data processing suite for protein informatics. Proteomes 9:15. doi:10.3390/proteomes9010015 PMC8006021 33806881

[45] Ting L, Rad R, Gygi SP, Haas W. 2011. MS3 eliminates ratio distortion in isobaric multiplexed quantitative proteomics. Nat Methods 8:937–940. doi:10.1038/nmeth.1714 PMC3205343 21963607

[46] Savitski MM, Mathieson T, Zinn N, Sweetman G, Doce C, Becher I, Pachl F, Kuster B, Bantscheff M. 2013. Measuring and managing ratio compression for accurate iTRAQ/TMT quantification. J Proteome Res 12:3586–3598. doi:10.1021/pr400098r 23768245

[47] Kanehisa M, Furumichi M, Sato Y, Kawashima M, Ishiguro-Watanabe M. 2023. KEGG for taxonomy-based analysis of pathways and genomes. Nucleic Acids Res 51:D587–D592. doi:10.1093/nar/gkac963 PMC9825424 36300620

[48] Korotkevich G, Sukhov V, Budin N, Shpak B, Artyomov MN, Sergushichev A. 2021. Fast gene set enrichment analysis. Bioinformatics. doi:10.1101/060012

[49] Virtanen P, Gommers R, Oliphant TE, Haberland M, Reddy T, Cournapeau D, Burovski E, Peterson P, Weckesser W, Bright J, et al.. 2020. SciPy 1.0: fundamental algorithms for scientific computing in Python. Nat Methods 17:261–272. doi:10.1038/s41592-019-0686-2 PMC7056644 32015543

[50] Waskom ML. 2021. Seaborn: statistical data visualization. JOSS 6:3021. doi:10.21105/joss.03021

[51] Hunter JD. 2007. Matplotlib: a 2D graphics environment. Comput Sci Eng 9:90–95. doi:10.1109/MCSE.2007.55

[52] Perez-Riverol Y, Bandla C, Kundu DJ, Kamatchinathan S, Bai J, Hewapathirana S, John NS, Prakash A, Walzer M, Wang S, Vizcaíno JA. 2025. The PRIDE database at 20 years: 2025 update. Nucleic Acids Res 53:D543–D553. doi:10.1093/nar/gkae1011 PMC11701690 39494541

[53] Edgar R, Domrachev M, Lash AE. 2002. Gene expression omnibus: NCBI gene expression and hybridization array data repository. Nucleic Acids Res 30:207–210. doi:10.1093/nar/30.1.207 PMC99122 11752295

