# Supplementary material for: The Agrobacterium fabrum efflux pump PecM serves as a critical metabolic relief valve to maintain cellular homeostasis
Source: mSphere. 2026 Oct 8;11(10):e00601-26. doi: 10.1128/msphere.00601-26 (PMC13711781; doi:10.1128/msphere.00601-26)
Supplement: Supplementary file 1 [file msphere.00601-26-s0001.pdf]

The *Agrobacterium fabrum* efflux pump PecM serves as a critical metabolic relief valve to maintain cellular homeostasis

*Arpita Ghosh,[1] Ahmed Al-Tohamy,[1] Fabrizio Donnarumma,[2] Anne Grove[1]**

[1]Department of Biological Sciences, Louisiana State University, Baton Rouge, LA 70803, USA
[2]Department of Chemistry, Louisiana State University, Baton Rouge, LA 70803, USA

<u>Contents</u>:

Supplemental Figure S1. Genomic context of select *pecS-pecM* gene pairs.

Supplemental Figure S2. PecM sequence conservation.

Supplemental Figure S3. Sensitivity to potentially toxic compounds and ions.

Supplemental Figure S4. Survey of phenotypes.

Supplemental Figure S5. Disruption of *pecM* does not affect chemotaxis.

Supplemental Figure S6. Response to environmental conditions.

Supplemental Figure S7. Density plot.

Expanded Materials and Methods

References

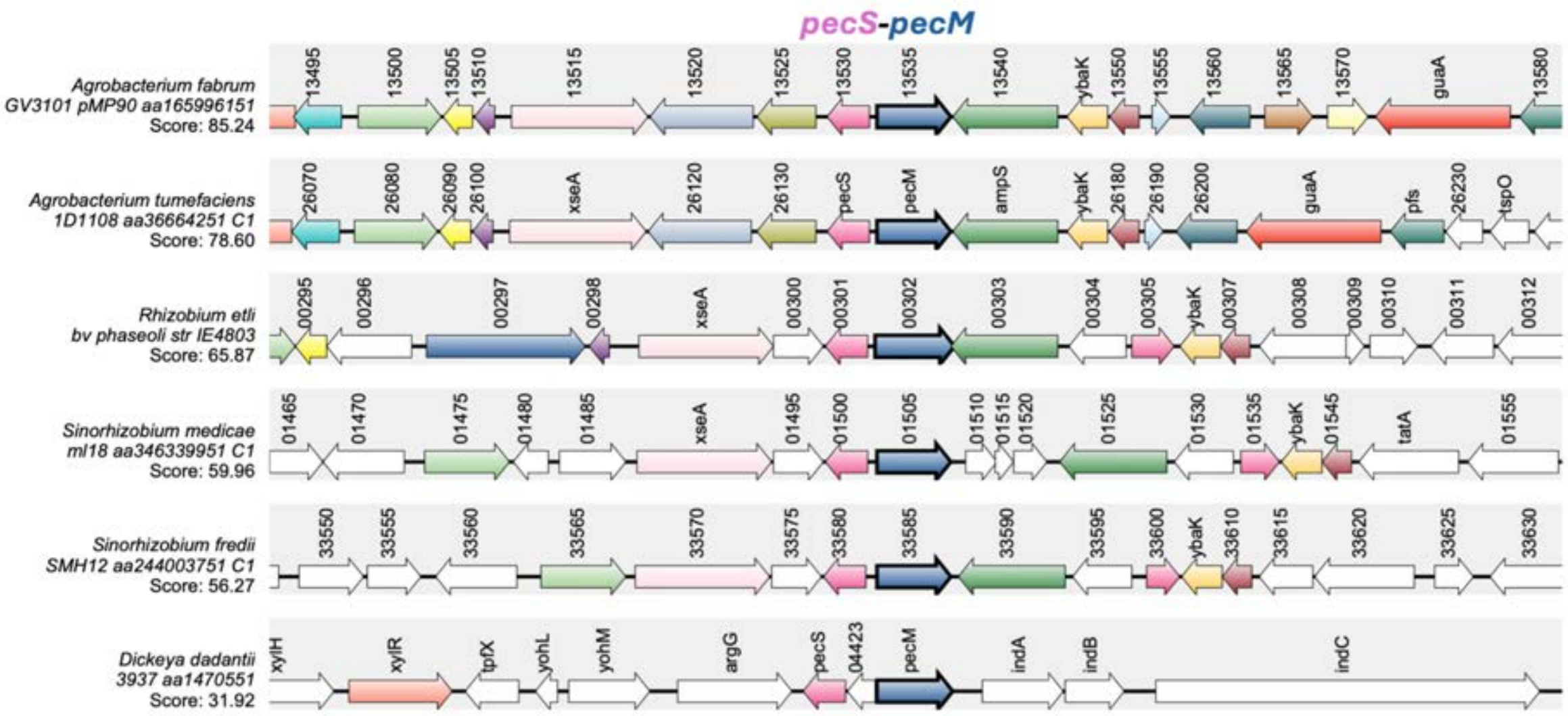

Supplemental Figure S1. Genomic context of select *pecS-pecM* gene pairs. The sequence of *A. fabrum* C58 PecM was submitted to the synteny web server SyntTax (1), and the genomic context of select α-proteobacteria as well as that of *Dickeya dadantii* 3937 was retrieved. The conserved *pecS-pecM* gene pair is at the center in pink and blue.

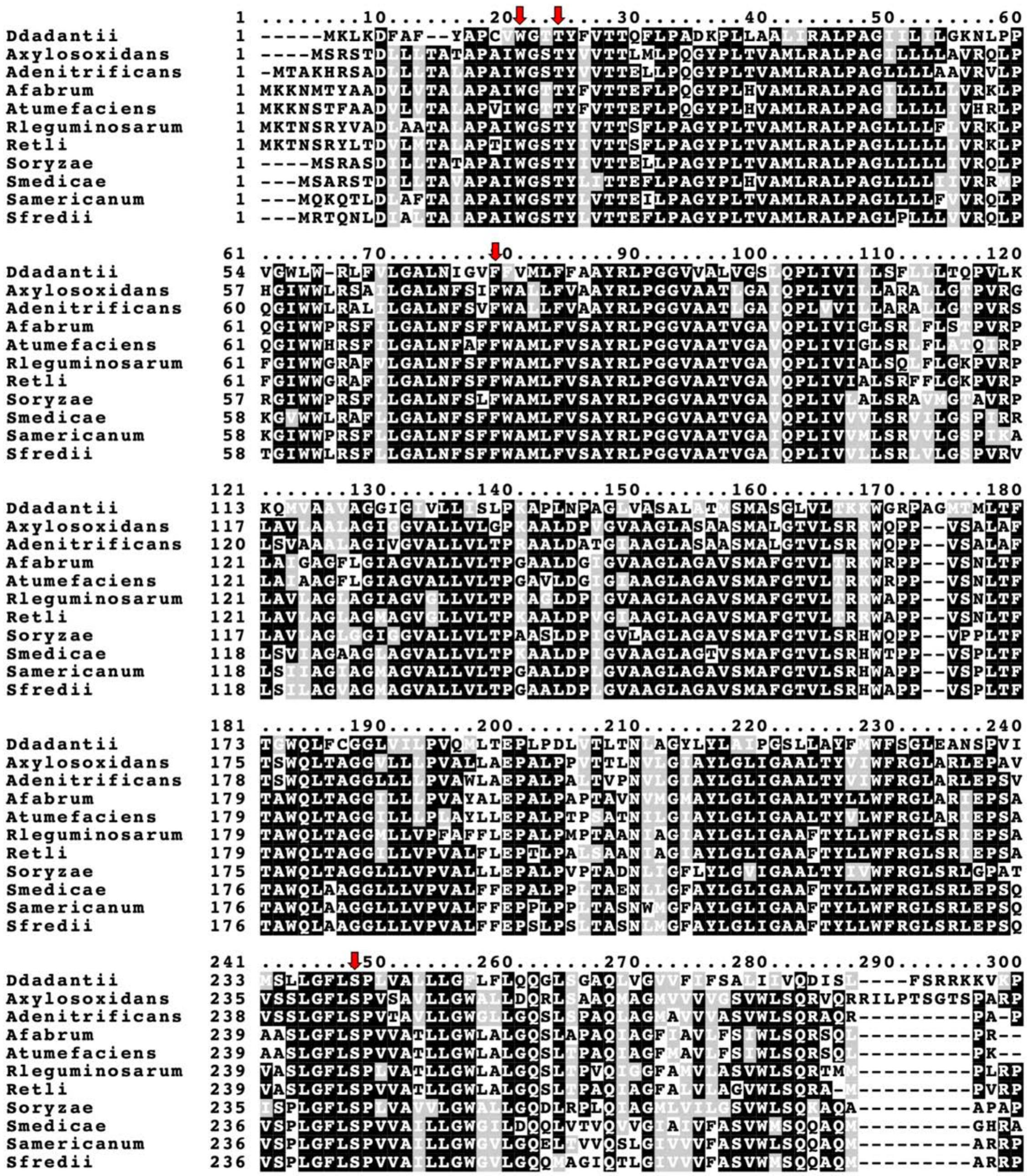

Supplemental Figure S2. PecM sequence conservation. The sequences of PecM from *D. dadantii*, two β-proteobacteria (*Achromobacter xylosoxidans* and *Achromobacter denitrificans*), and several α-proteobacteria (*A. fabrum*, *Agrobacterium tumefaciens*, *Rhizobium leguminosarum*, *Rhizobium etli*, *Sinorhizobium oryzae*, *Sinorhizobium medicae*, *Sinorhizobium americanum*, and *Sinorhizobium fredii*) were aligned using MUSCLE (2). Red arrows identify residues predicted to contact the *A. fabrum* PecM substrate 4-hydroxybenzaldehyde (Figure 1C).

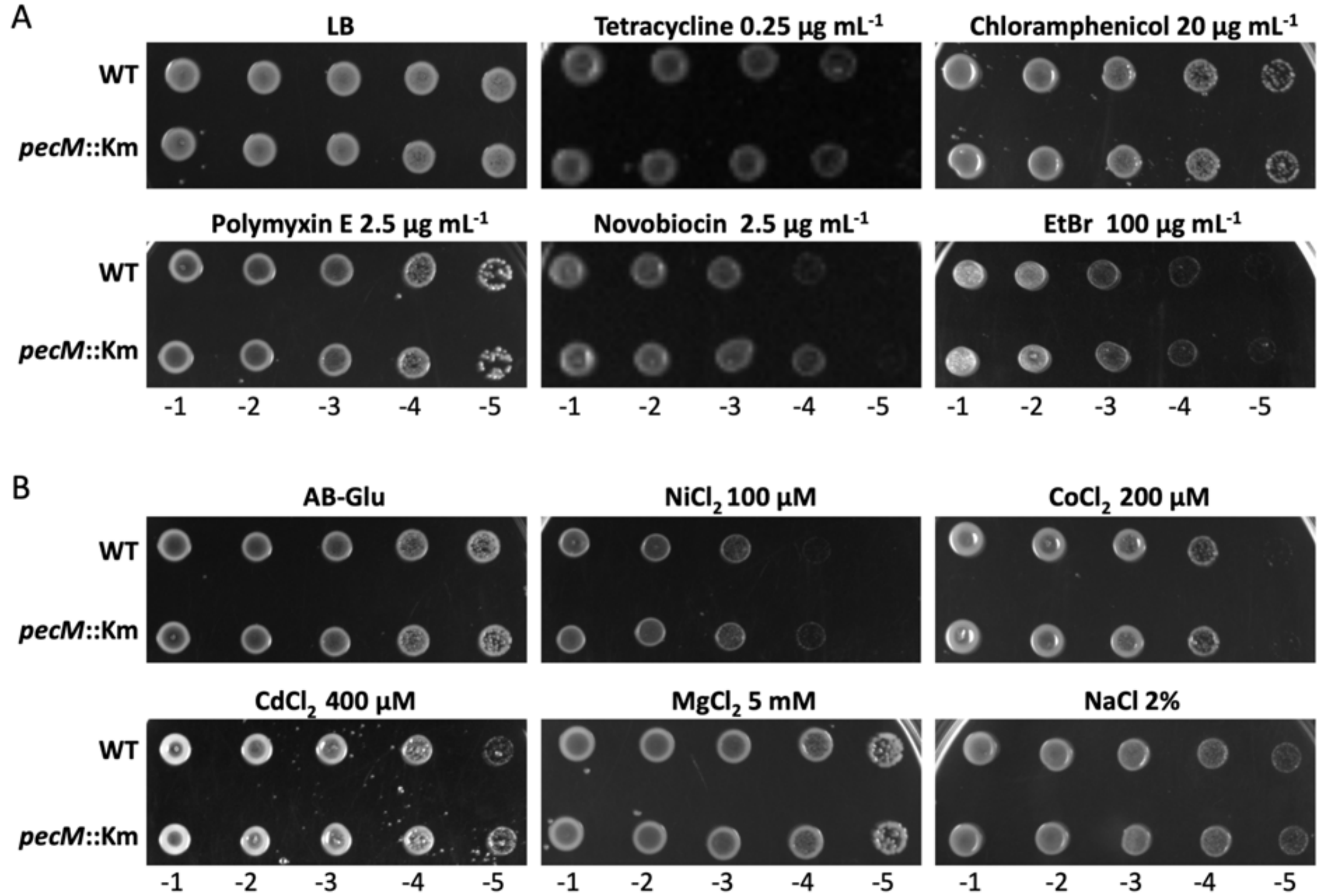

Supplemental Figure S3. Sensitivity to potentially toxic compounds and ions. A. Exponentially growing cells were 10-fold serially diluted (with the negative exponent of the 10-fold dilution factor provided beneath each panel), and 5 µL was spotted on LB-agar plates without or with the indicated compounds. Strains are identified at the left. Images were captured after 48 h incubation at 28°C. B. Exponentially growing cells were 10-fold serially diluted, and 5 µL was spotted on AB-glucose agar plates without or with the indicated compounds. Strains are identified at the left. Images were captured after 48 h incubation at 28°C. All images are representative of two biological replicates.

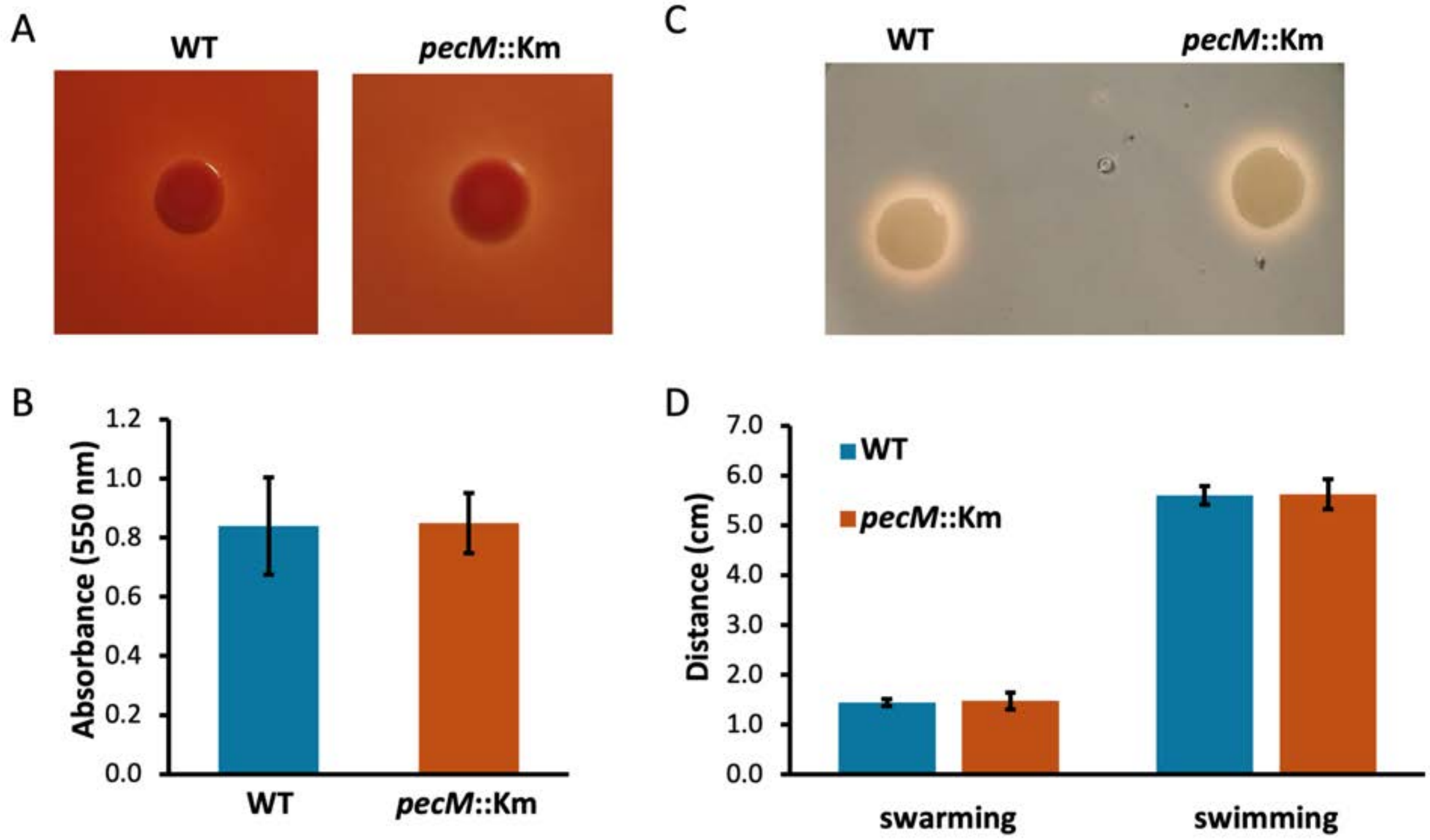

Supplemental Figure S4. Survey of phenotypes. A. Cells grown in LB for 48 hours at 28°C were diluted 1:100 in fresh media and grown to OD_{600}≈0.6. Five µL of culture was spotted on AB-glucose agar plates with 50 µg mL^{-1} Congo red dye. Plates were incubated at 28°C for 5 days before images were captured. B. One hundred µL of cells grown in LB for 48 hours at 28°C with shaking was added to 1 mL of fresh LB in polystyrene tubes. The tubes were incubated at 28°C under stationary conditions for 7 days. The culture was discarded, and the tube was washed twice with water to remove unattached cells. For crystal violet staining, 0.1% of 1 mL crystal violet in water was added to each tube, which were incubated at room temperature for 15 min. The tubes were washed with water three times and allowed to air dry. One mL of 30% acetic acid in water was added to each tube to release crystal violet bound to the biofilm. Absorbance of the solubilized crystal violet was measured at 550 nm. Data represent the average ± SD from three biological replicates. C. For detection of siderophore production, Chrome Azurol S (CAS) assay was performed as described previously (3). Cells grown in LB for 48 hours at 28°C was diluted to OD_{600}≈0.6. Five µL of culture was spotted at the center of a CAS agar plate. The plates were incubated at 28°C for 72 hours. The results are representative of three biological replicates. D. Cells grown in LB broth for 48 hours at 28°C were diluted 1:100 in fresh media and grown to OD_{600}≈0.6. Five µL of culture was spotted in the middle of swimming or swarming plates. Swim plates contained AB-glucose with 0.3% agar. The swarm plates contained 0.8% Difco nutrient broth, 0.5% glucose and 0.5% agar. For swimming and swarming, plates were incubated at 28°C for 3-5 days before images were captured, and the distance migrated was measured. Data represent the average ± SD from three biological replicates.

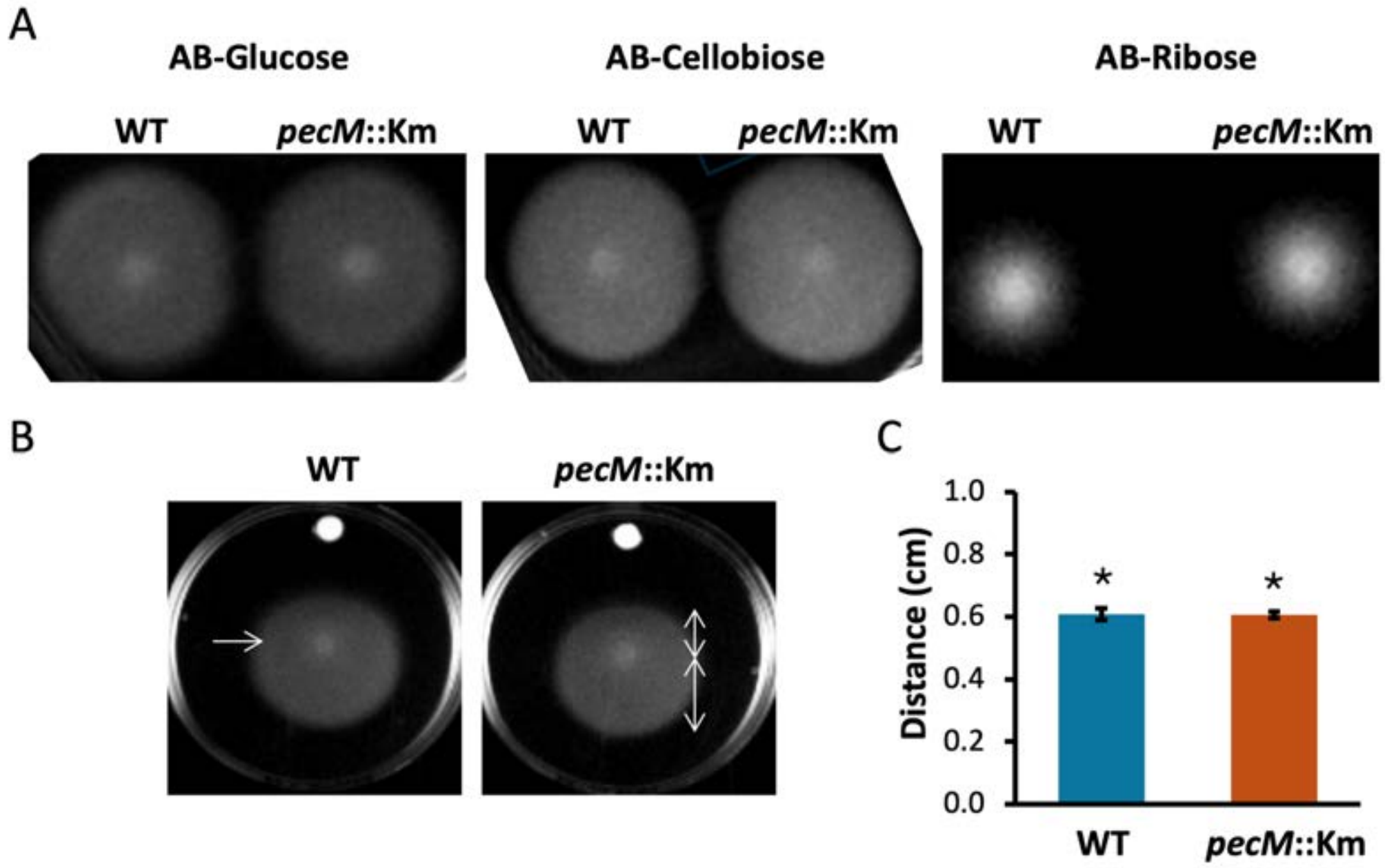

Supplemental Figure S5. Disruption of *pecM* does not affect chemotaxis. A. Chemotaxis towards the indicated sugars for WT and *pecM*::Km cells. Cells were spotted on AB minimal media plates supplemented with the indicated sugars. B. Chemotactic response to salicylate. Cells were spotted at the center of AB-glucose plates (lighter area at the center, marked with arrow in the left image), and 20 μL of 50 mM salicylate was spotted at the top edges (on white filter paper). C. Quantitation of the ratio of migration towards relative to away from salicylate; data represent the average of three biological replicates;. The distance migrated was measured from the point of inoculation to the edge of the colony near the salicylate spot or in the opposite direction (as identified by white arrows in panel B, right image); a ratio <1 indicates movement away from salicylate. Error bars represent SD. Asterisks indicate statistically significant difference from non-directional movement, which would be a T/A Ratio = 1.0; p<0.05.

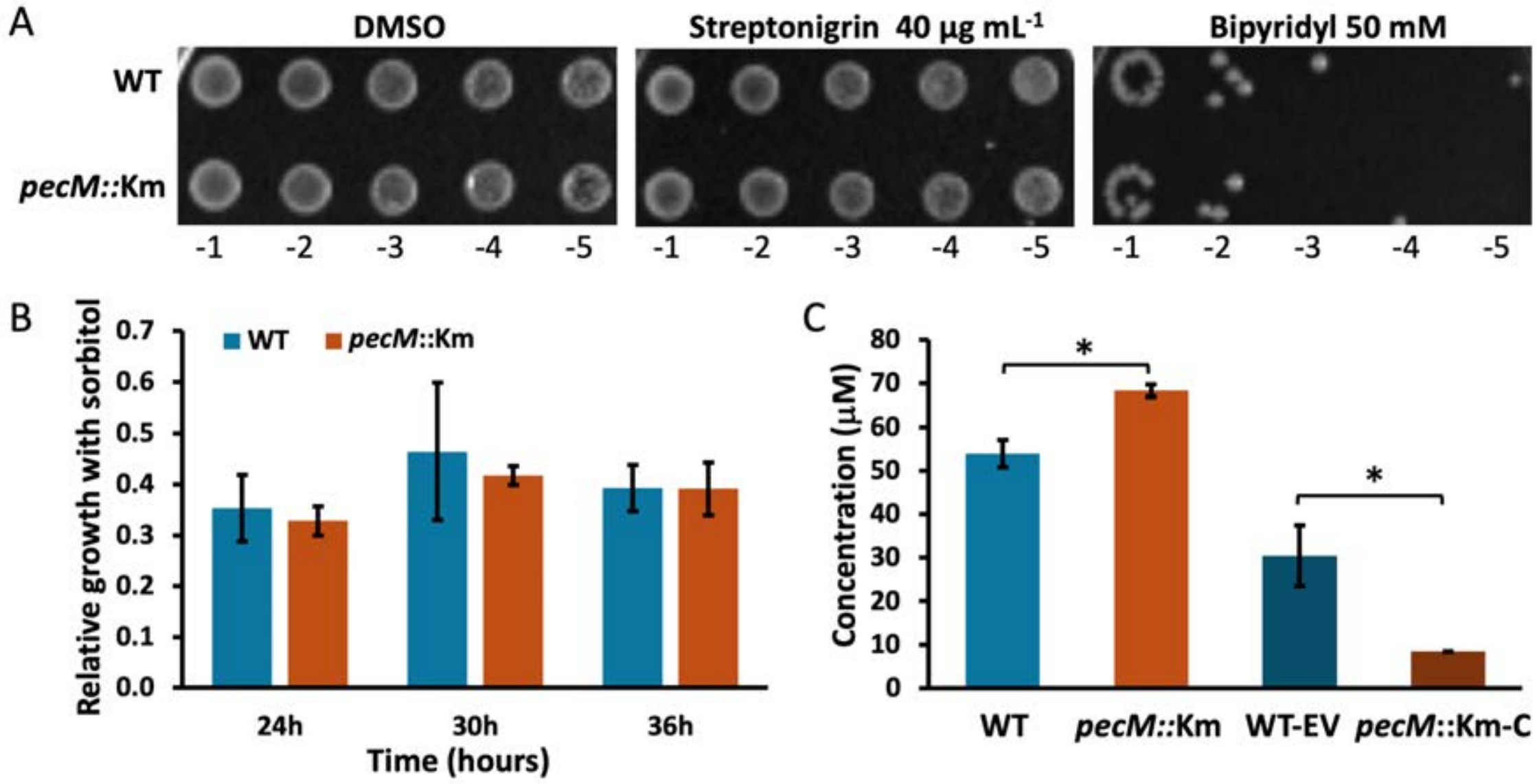

Figure S6. Response to environmental conditions. A. Cells grown in LB medium for 24 hours were diluted to OD_{600}≈0.1 with fresh LB. Cells were treated with 40 µg mL^{-1} streptonigrin or 50 mM 2,2'-bipyridyl for 3 hours at 28°C under shaking conditions. The control cells were treated with equivalent volume of dimethyl sulfoxide (DMSO). The cells were serially diluted and spotted on LB agar plates. The images were captured after 3 days incubation at 28°C. B. Cells were grown at 28°C in AB media with 0.2% sorbitol or 0.2% glucose. Data, representing three biological replicates, are presented as relative growth with sorbitol compared to glucose ±SD; the ratio <1 indicates that glucose is the favored carbon source. C. Intracellular levels of glutamate were determined using the Amplex™ Red assay kit, as described (4). Cells were grown in LB media at 28°C with shaking for 36 h before harvesting. The results are the mean ± SD from three independent experiments. The asterisks represent statistically significant differences based on Student's t-test, * $p < 0.05$.

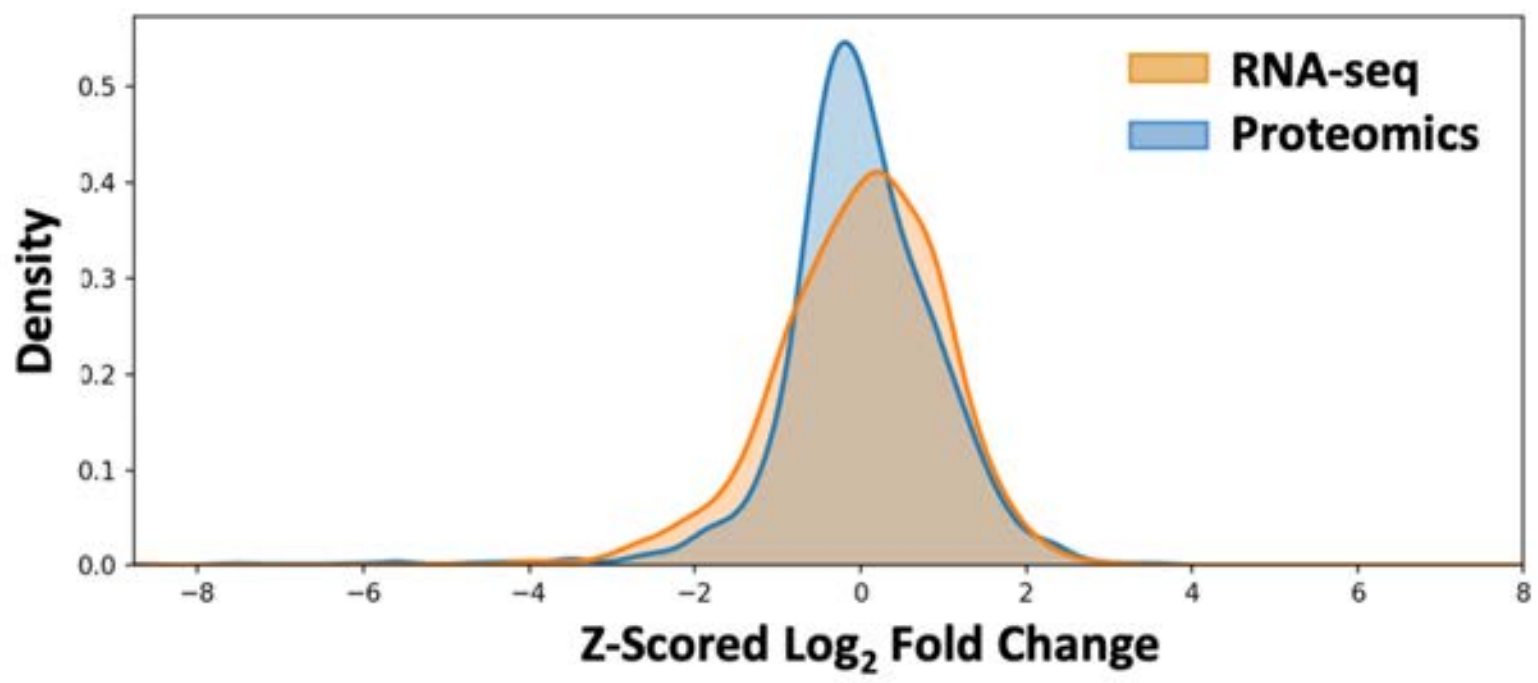

Figure S7. Density plot. Overlaid kernel density plots comparing the distribution of log_2 fold changes in RNA-seq (orange) and proteomics (blue) datasets. The x-axis represents normalized log_2 fold change, while the y-axis reflects the density of gene/protein occurrences.

Expanded Materials and Methods

RNA sequencing

Library preparation and high-throughput sequencing. Library preparation and high-throughput sequencing was performed by Novogene (https://www.novogene.com). Library preparation was achieved using the Illumina TruSeq Stranded mRNA Library Prep Kit. Ribosomal RNA was depleted, followed by ethanol precipitation. The RNA was fragmented, and the first strand of cDNA was synthesized using random hexamer primers. For synthesis of the second strand of cDNA, dTTP was used in place of dUTP. The library was processed by end repair, A-tailing, adapter ligation, and size selection. Uracil-Specific Excision Reagent (USER) enzyme digestion was performed, followed by amplification and purification. The libraries were quantified using Qubit and real-time PCR, and a bioanalyzer was used for determination of the size distribution. Libraries were pooled, and high-throughput sequencing was performed using a paired-end read, 150 bp configuration on the Illumina NextSeq 500 platform, and Fastq files were generated.

Bioinformatics analysis. The raw reads were assessed using FastQC (5). Skewer was used to remove adapter sequences, filter out ambiguous nucleotides, and trim low-quality bases (Phred score threshold of 20) (6). MultiQC was used to assess the quality of the processed data (7). The reference genome for *Agrobacterium fabrum* strain C58 was downloaded from NCBI RefSeq database (accession: GCF_000092025.1), and indexing was performed using SAMtools (8). High-quality reads were aligned to the indexed genome using HISAT2 (9). SAMtools was used to construct SAM and BAM files from the aligned reads for downstream processing. Gene-level quantification was achieved using FeatureCounts (10). Differential gene expression between the wild-type and *pecM*::Km was identified using DESeq2 (11). Genes were considered differentially expressed if they exhibited a log2-fold change of ≥1 or ≤-1 and if they met the statistical significance threshold with a false discovery rate (FDR) of <0.05. The adjusted *p*-value was corrected for multiple testing using the Benjamini-Hochberg procedure (12).

Quantitative mass spectrometry proteomics

Protein extraction, Tandem Mass Tag (TMT) labeling, and fractionation. The S-Trap (ProtiFi, Fairport, NY) sample processing method was used (13), with some modifications. Cells were grown in LB, and ~33 mL of bacterial culture was harvested at OD_{600}≈1.0 and collected by centrifugation at 17,800 × g for 5 min, harvesting cells from the same cultures used for RNA sequencing. After two washes with phosphate buffered saline (PBS), the cell pellets were stored at -80°C. The cell pellets were thawed on ice, and 1 mL of lysis buffer (0.5% sodium dodecylsulfate (SDS) in 100 mM tetraethylammonium bicarbonate (TEAB), pH 8.5) was added to each sample. Cells were lysed by sonication using a Branson SFX250 Sonifier at 20% output; a total of 4 cycles comprising 10 sec pulses with 1 min gap in between were implemented, keeping samples on ice. Homogenized samples were centrifuged at 8,600 × g for 5 min at 4°C, and the supernatants were transferred to a new tube. Protein concentration was determined using the Pierce™ bicinchoninic acid (BCA) Protein Assay Kit (Thermo Fisher Scientific).

Proteins (100 µg per sample) were reduced by incubation with 20 mM DTT for 5 min at 95 °C. Samples were cooled to room temperature, and proteins were alkylated with 40 mM iodoacetamide for 20 min in the dark, followed by acidification by addition of 27.5% phosphoric

acid until pH < 1. Wash buffer (5.5 volumes of 10% 100 mM TEAB, 90% methanol) was added to each sample, followed by vortexing. Each sample was added to a separate S-Trap column fitted with a microfuge tube and centrifuged at 4,000 × g for 30 sec, after which the columns were washed four times with 150 µL wash buffer. On-column protein digestion was performed with 2 µg/sample of LysC/Trypsin (Thermo Fisher Scientific) at 37°C overnight. The contents of the columns were then eluted step-wise with 40 µL each of elution buffer 1 (50 mM TEAB), elution buffer 2 (0.2% formic acid in water), and elution buffer 3 (50% acetonitrile in water), and eluates were combined in one tube. The eluted peptides were dried under vacuum, resuspended in 26 µL 500 mM TEAB (pH 8.5) and quantified using a Nanodrop spectrophotometer (Thermo Fisher Scientific).

TMTsixplex™ labeling was conducted as described (14); each of the six TMT reagents were dissolved in 41 µL of anhydrous acetonitrile (ACN). Ten µL of each TMT reagent was added to 35 µg of peptide, the volume was adjusted to 36 µL using TEAB, and samples were incubated for 1 hour at room temperature. Two µL of 5% hydroxylamine was then added to quench reactions. Equal amounts (5 µg) of labeled peptides from each condition were pooled and stored at –20°C. After labeling, the six TMT-labeled peptide samples (three representing wild-type and three representing *pecM*::Km) were pooled and fractionated into six fractions using strong cation exchange stage tips (AttractSPE Disk, Affinisep, Le Houlme, Normandy, France) (14). The tips were conditioned twice with 200 µL of ACN, followed by equilibration with 200 µL of 0.1% formic acid in water. Peptides (30 µg total) were loaded onto the tip and washed twice with 200 µL of 0.2% formic acid in water. Fractionation was performed using a stepwise elution with ammonium acetate (NH_4OAc, pH 3.0) in 0.1% formic acid (FA), with the following conditions (200 µL of each solution): Fraction 1: 50 mM NH_4OAc, 20% ACN, 0.5% FA; Fraction 2: 75 mM NH_4OAc, 20% ACN, 0.5% FA; Fraction 3: 125 mM NH_4OAc, 20% ACN, 0.5% FA; Fraction 4: 200 mM NH_4OAc, 20% ACN, 0.5% FA; Fraction 5: 300 mM NH_4OAc, 20% ACN, 0.5% FA; Fraction 6: 5% ammonium hydroxide, 80% CAN. (14) These 6 fractions were dried and resuspended in 10 µL 0.1% formic acid for injection.

Data acquisition. A Q-Exactive orbitrap mass spectrometer (Thermo Scientific, Waltham, MA) coupled to a Vanquish Neo liquid chromatography system (Thermo Scientific) was used for mass spectrometry. A binary mobile phase system was used for both sample loading and trapping and for analytical separation. Sample injection was set to 5 µL. The mobile phase composition was as follows: A = LC-MS grade H_2O with 0.1% formic acid and B = 80% acetonitrile with 0.1% formic acid. For sample loading, a capillary carrying the peptides was connected to an Acclaim PepMap 100 C18 trap cartridge (0.3 x 5 mm, 5 µm particle size, 100 Å pore size) and peptides were transferred using 100 µL of a mobile phase of 100% A at a flow rate of 20 µL min^{-1}. Separation was carried out on the Acclaim PepMap 100 C18 nanoLC column (0.075 × 250 mm, 3 µm particle size, 100 Å pore size) using a linear gradient from 6% to 32% mobile phase B over the course of 120 minutes at a flow rate of 300 nL min^{-1}. The mass spectrometer was operated in data-dependent acquisition mode with a loop count of 10. Additional MS parameters were as follows: resolution = 70,000, automatic gain control (AGC) = 1×10^6, scan range = 300-1600 *m/z* and maximum injection time = 30 ms. The data-dependent acquisition parameters were as follows: resolution = 35,000, AGC target = 5×10^4, isolation window = 1.4 *m/z*, maximum injection time = 50 ms and normalized collision energy = 30. Additional settings included: dynamic

exclusion = 90 seconds, peptide match = preferred, excluded isotopes = on, charge exclusion = unassigned +7, +8 and >+8 charges, and maximum AGC target = 500, which resulted in an intensity threshold of 10,000.

Proteomics data analysis. Raw mass spectra were subjected to data processing using Proteome Discoverer 2.4, SP1 (PD) (15). Peak picking, spectral alignment, and peptide identification was achieved using the SEQUEST HT and MASCOT (Matrix Science, London, UK) search engines. The acquired spectra were matched against the *Agrobacterium fabrum* C58 (ATCC 33970) protein database obtained from UniProt (5,344 entries) (16). Spectra were searched with a mass tolerance of 10 ppm at the parent ion level and 0.02 *m/z* at the fragment ion level. Percolator was used to calculate false discovery rates (FDR) at the peptide levels, while the native Proteome Discoverer protein FDR validator was used to calculate FDR at the protein level. Only peptides and proteins with FDR < 0.1 were used. Only proteins for which at least two peptides were identified, of which one unique, were included. TMT reporter ion intensities were used for quantification of protein abundances. The protein ratio calculation was set to "protein abundance based", and the hypothesis test for p-value generation was set to ANOVA (individual proteins). No imputation mode was employed for resampling of missing values. Multiple hypothesis correction was applied at the protein level using the Benjamini–Hochberg procedure, as implemented in PD, to control for the false discovery rate. Proteins were considered significantly differentially expressed based on an absolute log2-fold change ≥0.4 and a statistical significance threshold of p-value < 0.05.

Data visualization. A Principal Component Analysis (PCA) was conducted using the PCA function from the sklearn.decomposition module in Python (17). The proteomic data were standardized using z-score normalization, where each protein abundance value was transformed by subtracting the mean and dividing by the standard deviation across all samples. The PCA results were visualized using matplotlib (18). The volcano plot was generated using the Plotly Express library in Python (19). KEGG pathway enrichment was conducted using rank-based Gene Set Enrichment Analysis (GSEA) using custom R and Python scripts. All confidently detected proteins were first ranked according to their $\log_2$ fold change between conditions, without imposing any fold change or significance cutoff. Statistical significance was determined using permutation based enrichment scores, and p-values were calculated based on the null distribution generated by gene or protein label permutations. This rank-based approach allows detection of pathway level trends resulting from coordinated changes for multiple pathway members, even when individual genes or proteins do not meet the otherwise imposed thresholds. KEGG pathway enrichment outputs were visualized using bubble plots constructed in R with the ggplot2 package (20). Analyses were performed in R version 4.6.0 (21).

Integration of RNA-seq and proteomics data

To integrate RNA-Seq and proteomics data, curated annotations from the UniProt database were used to map corresponding genes and proteins. To enable a comparison of expression changes, $\log_2$-fold change values from both datasets were standardized using z-score normalization via the StandardScaler function from the scikit-learn library (17). For each dataset, the mean and standard deviation were calculated across all matched genes, and each gene's value was centered and scaled relative to these parameters. This transformation expresses each gene's

response in units of standard deviation from the mean, thereby accounting for differences in variance and dynamic range between RNA-seq and proteomic measurements.

To integrate transcriptomic and proteomic signals, a composite ranking metric was constructed that captures both the direction and magnitude of regulation across datasets. Specifically, for each gene, a score was defined as:

$$\mathrm{sgn}(RNA_z + Protein_z) \times \sqrt{RNA_z^2 + Protein_z^2}$$

This formulation preserves the overall direction of regulation while incorporating the combined strength of the transcriptomic and proteomic responses. Compared to simple averaging, this approach avoids signal attenuation and ensures that genes exhibiting strong coordinated changes are prioritized in downstream analyses.

Gene Set Enrichment Analysis (GSEA) was performed using this ranked list across the full set of matched genes, rather than applying arbitrary thresholds to pre-filter the data. This approach allows for the detection of coordinated pathway-level changes driven by both strong and moderate gene-level signals. KEGG pathway annotations were used to define gene sets, and pathways were filtered to include those with sufficient representation in the ranked dataset to ensure robust enrichment statistics.

Enrichment scores (ES) and normalized enrichment scores (NES) were calculated for each pathway, and statistical significance was assessed using permutation-based p-values. Leading-edge subsets were extracted to identify the genes contributing most strongly to the enrichment signal for each pathway.

The relationship between transcript and protein abundance changes was assessed through Pearson correlation analysis, with correlation coefficients and associated *p*-values calculated using the SciPy library (22). Visualization was performed using the seaborn and matplotlib libraries.(18, 23) Detailed information on statistical methods were reported previously (14).

For biological interpretation, genes were additionally categorized based on the direction of their normalized transcriptomic and proteomic responses into four regulatory groups: concordant upregulated, concordant downregulated, discordant with transcript upregulation and protein downregulation, and discordant with transcript downregulation and protein upregulation. While enrichment analysis was conducted on the full ranked dataset, these categories were used to assess the contribution of specific regulatory patterns to enriched pathways, including their representation within leading-edge gene sets.

References

1. Oberto J. 2013. SyntTax: a web server linking synteny to prokaryotic taxonomy. BMC Bioinformatics 14:4.
2. Edgar RC. 2004. MUSCLE: a multiple sequence alignment method with reduced time and space complexity. BMC Bioinformatics 5:113.
3. Louden BC, Haarmann D, Lynne AM. 2011. Use of Blue Agar CAS Assay for Siderophore Detection. J Microbiol Biol Educ 12:51-3.
4. Ghosh A, Grove A. 2025. The *Agrobacterium fabrum* efflux pump PecM is produced in response to the plant exudate 4-hydroxybenzaldehyde to avoid disruption of central metabolism. J Bacteriol 207:e0015025.
5. Andrews S. 2010. FastQC: A Quality Control Tool for High Throughput Sequence Data. http://www.bioinformatics.babraham.ac.uk/projects/fastqc/. Accessed
6. Jiang H, Lei R, Ding SW, Zhu S. 2014. Skewer: a fast and accurate adapter trimmer for next-generation sequencing paired-end reads. BMC Bioinformatics 15:182.
7. Ewels P, Magnusson M, Lundin S, Kaller M. 2016. MultiQC: summarize analysis results for multiple tools and samples in a single report. Bioinformatics 32:3047-8.
8. Li H, Handsaker B, Wysoker A, Fennell T, Ruan J, Homer N, Marth G, Abecasis G, Durbin R, Genome Project Data Processing S. 2009. The Sequence Alignment/Map format and SAMtools. Bioinformatics 25:2078-9.
9. Kim D, Paggi JM, Park C, Bennett C, Salzberg SL. 2019. Graph-based genome alignment and genotyping with HISAT2 and HISAT-genotype. Nat Biotechnol 37:907-915.
10. Liao Y, Smyth GK, Shi W. 2014. featureCounts: an efficient general purpose program for assigning sequence reads to genomic features. Bioinformatics 30:923-30.
11. Love MI, Huber W, Anders S. 2014. Moderated estimation of fold change and dispersion for RNA-seq data with DESeq2. Genome Biol 15:550.
12. Benjamini Y, Drai D, Elmer G, Kafkafi N, Golani I. 2001. Controlling the false discovery rate in behavior genetics research. Behav Brain Res 125:279-84.
13. Zougman A, Selby PJ, Banks RE. 2014. Suspension trapping (STrap) sample preparation method for bottom-up proteomics analysis. Proteomics 14:1006-1010.
14. Al-Tohamy A, Donnarumma F, Grove A. 2025. Transcriptome-proteome profiling in *Burkholderia thailandensis* during the transition from exponential to stationary phase. J Proteome Res 24:4082-4097.
15. Orsburn BC. 2021. Proteome Discoverer-A Community Enhanced Data Processing Suite for Protein Informatics. Proteomes 9:15.
16. The UniProt Consortium. 2023. UniProt: the Universal Protein Knowledgebase in 2023. Nucleic Acids Res 51:D523-D531.
17. Pedregosa F, Varoquaux G, Gramfort A, Michel V, Thirion B, Grisel O, Blondel M, Prettenhofer P, Weiss R, Dubourg V, Vanderplas J, Passos A, Cournapeau D, Brucher M, Perrot M, Duchesnay E. 2011. Scikit-learn: Machine learning in python. J Mach Learn Res 12:2825-2830.
18. Hunter JD. 2007. Matplotlib: A 2D graphics environment. Comput Sci Eng 9:90-95.
19. Plotly Technologies Inc. 2015. Collaborative data science., *on* Plotly Technologies Inc. https://plot.ly. Accessed

20. Wickham H. 2016. ggplot2: Elegant Graphics for Data Analysis. Springer, New York.
21. R Core Team. 2020. R: A Language and Environment for Statistical Computing. R Foundation for Statistical Computing, Vienna, Austria.
22. Virtanen P, Gommers R, Oliphant TE, Haberland M, Reddy T, Cournapeau D, Burovski E, Peterson P, Weckesser W, Bright J, van der Walt SJ, Brett M, Wilson J, Millman KJ, Mayorov N, Nelson ARJ, Jones E, Kern R, Larson E, Carey CJ, Polat I, Feng Y, Moore EW, VanderPlas J, Laxalde D, Perktold J, Cimrman R, Henriksen I, Quintero EA, Harris CR, Archibald AM, Ribeiro AH, Pedregosa F, van Mulbregt P, SciPy C. 2020. SciPy 1.0: fundamental algorithms for scientific computing in Python. Nat Methods 17:261-272.
23. Waskom ML. 2021. Seaborn: statistical data visualization. J Open Source Softw 6:3021.
