# Supplementary material for: The Agrobacterium fabrum efflux pump PecM serves as a critical metabolic relief valve to maintain cellular homeostasis
Source: mSphere. 2026 Oct 8;11(10):e00601-26. doi: 10.1128/msphere.00601-26 (PMC13711781; doi:10.1128/msphere.00601-26)
Supplement: Supplementary file 2 [file msphere.00601-26-s0002.pdf]

The *Agrobacterium fabrum* efflux pump PecM serves as a critical metabolic relief valve to maintain cellular homeostasis

Arpita Ghosh,[1] *Ahmed Al-Tohamy,*[1] *Fabrizio Donnarumma,*[2] *Anne Grove*[1]*

[1]Department of Biological Sciences, Louisiana State University, Baton Rouge, LA 70803, USA
[2]Department of Chemistry, Louisiana State University, Baton Rouge, LA 70803, USA

Supplemental Table Legends:

Table S1. Differentially expressed genes (DEGs).

Table S2. Proteins detected by proteomics.

Table S3. Peptides detected by proteomics.

Table S4. Summary of significantly enriched KEGG pathways from Gene Set Enrichment Analysis (GSEA) for pairs of accumulating DEGs and DEPs.

Table S5. Summary of significantly enriched KEGG pathways from Gene Set Enrichment Analysis (GSEA) for pairs of depleted DEGs and DEPs.

Table S6. Summary of significantly enriched KEGG pathways from Gene Set Enrichment Analysis (GSEA) for pairs of DEGs and DEPs in which the DEGs are depleted and the DEPs accumulate.

Table S7. Summary of significantly enriched KEGG pathways from Gene Set Enrichment Analysis (GSEA) for pairs of DEGs and DEPs in which the DEGs accumulate and the DEPs are depleted.
